# BISCUIT: an efficient, standards-compliant tool suite for simultaneous genetic and epigenetic inference in bulk and single-cell studies

**DOI:** 10.1093/nar/gkae097

**Published:** 2024-02-27

**Authors:** Wanding Zhou, Benjamin K Johnson, Jacob Morrison, Ian Beddows, James Eapen, Efrat Katsman, Ayush Semwal, Walid Abi Habib, Lyong Heo, Peter W Laird, Benjamin P Berman, Timothy J Triche, Hui Shen

**Affiliations:** Department of Epigenetics, Van Andel Institute, Grand Rapids, MI 49503, USA; Department of Epigenetics, Van Andel Institute, Grand Rapids, MI 49503, USA; Department of Epigenetics, Van Andel Institute, Grand Rapids, MI 49503, USA; Department of Epigenetics, Van Andel Institute, Grand Rapids, MI 49503, USA; Department of Epigenetics, Van Andel Institute, Grand Rapids, MI 49503, USA; Department of Developmental Biology and Cancer Research, Institute for Medical Research Israel-Canada, Faculty of Medicine, The Hebrew University of Jerusalem, Jerusalem 9112102, Israel; Department of Epigenetics, Van Andel Institute, Grand Rapids, MI 49503, USA; Department of Epigenetics, Van Andel Institute, Grand Rapids, MI 49503, USA; Department of Epigenetics, Van Andel Institute, Grand Rapids, MI 49503, USA; Department of Epigenetics, Van Andel Institute, Grand Rapids, MI 49503, USA; Department of Developmental Biology and Cancer Research, Institute for Medical Research Israel-Canada, Faculty of Medicine, The Hebrew University of Jerusalem, Jerusalem 9112102, Israel; Department of Epigenetics, Van Andel Institute, Grand Rapids, MI 49503, USA; Department of Epigenetics, Van Andel Institute, Grand Rapids, MI 49503, USA

## Abstract

Data from both bulk and single-cell whole-genome DNA methylation experiments are under-utilized in many ways. This is attributable to inefficient mapping of methylation sequencing reads, routinely discarded genetic information, and neglected read-level epigenetic and genetic linkage information. We introduce the BISulfite-seq Command line User Interface Toolkit (BISCUIT) and its companion R/Bioconductor package, biscuiteer, for simultaneous extraction of genetic and epigenetic information from bulk and single-cell DNA methylation sequencing. BISCUIT’s performance, flexibility and standards-compliant output allow large, complex experimental designs to be characterized on clinical timescales. BISCUIT is particularly suited for processing data from single-cell DNA methylation assays, with its excellent scalability, efficiency, and ability to greatly enhance mappability, a key challenge for single-cell studies. We also introduce the epiBED format for single-molecule analysis of coupled epigenetic and genetic information, facilitating the study of cellular and tissue heterogeneity from DNA methylation sequencing.

## Graphical Abstract

**Graphical Abstract:**
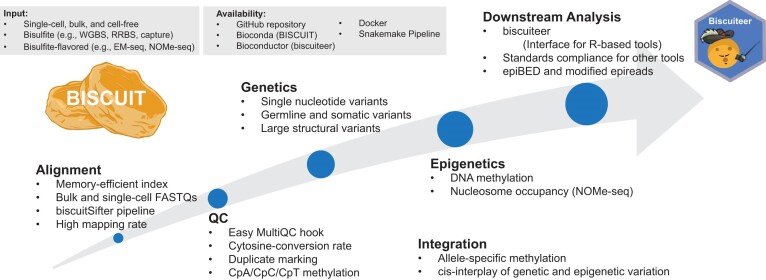

## Introduction

DNA methylation, commonly occurring in CpG dinucleotides, is an important epigenetic mark (1). It is robust to storage conditions and can be recovered from fresh frozen and formalin-fixed, paraffin-embedded (FFPE) samples, making it an excellent clinical biomarker to inform on disease etiology, diagnostics, and prognostics (2). Common methods to profile DNA methylation use sodium bisulfite treatment followed by PCR amplification to convert the difference between a methylated cytosine (mC) and an unmethylated cytosine (C) into a genetic difference (mC to C and C to T) (3). This can be followed by array-based or sequencing approaches for genome-scale interrogation of DNA methylation. Whole-genome bisulfite sequencing (WGBS) offers the most extensive genome coverage, allowing base-pair resolution of DNA methylation status. Recent methods, including Enzymatic Methyl-Seq (EM-seq) (4) and TET-assisted pyridine borane sequencing (TAPS) (5), employ enzymes instead of sodium bisulfite for the same conversion. In addition, NOMe-seq (nucleosome occupancy and methylome sequencing) simultaneously interrogates genome-wide nucleosome positioning, along with cytosine methylation, through the use of the GpC methyltransferase, M.CviPI (6). In the past decade, these WGBS and WGBS-like (broadly referred to as WGBS throughout) approaches have been adapted and applied to single cells to dissect epigenetic heterogeneity found within tissues (7), which has presented new analytical challenges. Current pipelines and tools developed for bulk experiments often cannot be directly used for single-cell experiments and align paired-end reads as single-end reads, thus losing the correlation between read pairs. Existing tools often require long processing times and have high memory/storage demands. These drawbacks are exacerbated when scaling to hundreds or thousands of cells. In addition, alignment of reads from single-cell experiments is particularly challenging due to higher error rates by the polymerase used and potential chimeric reads introduced. The sparsity of measuring millions of methylation sites throughout the genome in single cells also calls for higher alignment efficiency (8).

As the interplay of genetics and epigenetics has become increasingly appreciated (9,10), particularly in cancer (11,12), large-scale genomic studies have often included both whole-genome sequencing (WGS) and DNA methylation experiments, thus increasing the per-sample cost. It is underappreciated that the presence of genetic information in WGBS experiments can be utilized for detection of single-nucleotide polymorphisms (SNPs) and structural variation (SV) (13,14). Tools exist for SNP detection, exemplified by Bis-SNP (13). However, Bis-SNP relies on the Genome Analysis Toolkit (GATK) (15), requiring additional tool installation and computational overhead, increasing overall analysis time. In addition, no such tools exist for large SVs.

We present BISCUIT (BISulfite-seq Command line User Interface Toolkit), a multi-threaded, repeat- and cytosine-conversion-aware WGBS aligner whose performance, flexibility, standards-compliant output formats, and support toolchain allows large, complex experimental designs to be characterized on clinically relevant timescales. This framework allows for recovery of genetic and epigenetic information, with output formats that readily integrate with downstream tools to enable broad clinical research applications. Its companion R/Bioconductor package, biscuiteer, facilitates out-of-core-analysis of large WGBS experiments in resource-limited environments.

## Materials and methods

Materials and methods used to generate data are described below. More details about the methods can be found in the Supplemental Methods. For examples of the code used, see https://github.com/huishenlab/biscuit_paper_code.

### Alignment validation

Data for the alignment validation are from ten TruSeq Methyl Capture EPIC datasets available on SRA (Supplementary Table S1) (16). Read trimming was applied using TrimGalore! (https://github.com/FelixKrueger/TrimGalore, version 0.6.6 with cutadapt version 3.2). The manufacturer's manifest (https://support.illumina.com/downloads/truseq-methyl-capture-epic-manifest-file.html) for the on-target region set was downloaded and lifted over from hg19 to hg38 using the UCSC site: https://genome.ucsc.edu/cgi-bin/hgLiftOver.

Each dataset was aligned to hg38 with no contigs. Genome indexes were created for each aligner following the specified indexing protocol. The BISCUIT (version 1.2.1), Bismark (version 0.24.0) (17), BSBolt (version 1.6.0) (18), bwa-meth (version 0.2.6) (19), and gemBS (version 4.0.4) (20) pipelines follow best practices for analysis with each toolkit (Supplementary Figure S1).

The BISCUIT pipeline consisted of two primary steps: (i) alignment, duplicate marking with dupsifter (version 1.1.1, the ‘biscuitSifter’ pipeline described below) (21), and coordinate sorting and indexing with samtools (version 1.17) (22) and (ii) methylation extraction with biscuit pileup, compression and indexing with bgzip and tabix (version 1.17) (23), and converting to BED format and compressing with biscuit vcf2bed and gzip (version 1.12).

The Bismark pipeline used Bowtie2 (version 2.5.1) for the alignment, removed duplicates using deduplicate_bismark, and extracted methylation with bismark_methylation_extractor.

The BSBolt pipeline had three primary steps: (i) alignment with BSBolt, (ii) duplicate marking with samtools and (iii) methylation extraction with BSBolt.

The bwa-meth pipeline performed the alignment with bwa-meth, coordinate sorted and indexed with samtools, marked duplicates with Picard MarkDuplicates (version 2.27.5, https://broadinstitute.github.io/picard/), and extracted methylation with MethylDackel (version 0.6.1, https://github.com/dpryan79/MethylDackel).

gemBS is a self-contained pipeline, with all needed dependencies provided with gemBS when installing. When running each subcommand of the suggested pipeline (in order: prepare, map, call, and extract), gemBS will perform all necessary calls to other tools.

The number of reads for each sample was taken from the read 1 FASTQ file. The number of mapped and optimally mapped reads were calculated using samtools. Mapped reads include all reads except those that are flagged as secondary or supplementary. Optimally mapped reads also require the mapping quality score (MAPQ) be $ \ge$ 40. Reads that were on-target were determined by intersecting the aligned BAM with the manufacturer's manifest file using bedtools (version 2.30.0) (24).

### Speed benchmarking

Data for the speed benchmarking are available on SRA and come from human, mouse, and zebrafish samples across different tissue and disease states using both traditional WGBS and the more recent EM-seq (Supplementary Table S2) (4,25–29). For each dataset, the FASTQ files were subsampled to 1, 5, 10, 25, 50, 100 and 250 million reads using seqtk (version 1.3-r113-dirty, https://github.com/lh3/seqtk). It should be noted for the zebrafish datasets that multiple samples were combined for individual datasets in order to reach a sizeable number of reads to subsample. Further, one zebrafish dataset, even after combining, only had enough reads to subsample up to 50 million reads. Apart from the TCGA samples, which were already trimmed, read trimming was applied using the same process as performed in the alignment validation.

Human datasets were aligned to hg38 with no contigs, while mouse datasets were aligned to mm10 with no contigs. Zebrafish datasets were aligned to z11 with contigs. Genome indexes were created for each tool following the specified indexing protocol. The time to create the genome indexes was not included in the benchmarking times presented. For each time point collected, GNU time (version 1.9) was used.

For speed benchmarking, the same pipelines described for the alignment validation were used. To perform a fair comparison throughout, we made our best effort to adhere to best practices outlined for the other tools in publicly available pipelines (nf-core for Bismark and bwa-meth: https://nf-co.re/methylseq/2.6.0) or their respective online documentation (BSBolt version 1.6.0 documentation: https://bsbolt.readthedocs.io/en/latest/ and gemBS v4.0 User Guide: http://statgen.cnag.cat/gemBS/UserGuide/_build/html/index.html). Wherever a pipeline showed the use of multithreading, we used 30 threads, both for the tool itself and any third-party tools. In general, the alignment time is the amount of time needed to get a BAM that is duplicate marked, sorted, and indexed, while the methylation extraction time is the time to extract methylation from the sorted BAM. The end-to-end time is the sum of the alignment and methylation extraction times.

The BISCUIT alignment time is sum of the time to run the biscuitSifter pipeline (BISCUIT, dupsifter and samtools) and the indexing time. The methylation extraction time is the sum of the pileup, bgzip, tabix and vcf2bed times.

The Bismark alignment time is the sum of the align and deduplication times, while the methylation extraction time is the time to run bismark_methylation_extractor. Bismark does not need to be sorted to extract methylation, so sorting and indexing was not included.

The BSBolt alignment time is the time to align with BSBolt and fix mates, sort, mark duplicates and index with samtools. The methylation extraction time is the time to call methylation with BSBolt.

The bwa-meth alignment time is the sum of the alignment, sort, duplicate marking, and two indexing times. One index is needed for marking duplicates with Picard, while the second is needed to index the duplicate marked BAM output from Picard. The methylation extraction time is the time to extract methylation with MethylDackel.

The gemBS alignment time is the time to prepare and map with gemBS. The methylation extraction time is the time to call and extract methylation.

### Single-cell WGBS alignments

Two different single-cell WGBS datasets were used for this analysis. 249 single cells (153 human cells and 96 mouse cells) were taken from snmC-seq2 (GEO accession number GSE112471) (30) and 49 mouse cells from the Smallwood *et al.* protocol paper (GEO accession number GSE56879, only oocytes and embryonic stem cells were used) (31) were downloaded from SRA. The Smallwood *et al.* data was not trimmed, while the snmC-seq2 data was trimmed to remove barcodes and Adaptase bases from reads 1 and 2 with cutadapt (32) and then compressed with pigz.

The BISCUIT pipeline for both snmC-seq2 and Smallwood *et al.* followed the same pipeline used in the alignment validation with two small alterations (aligning in non-directional mode and loosening the depth restriction for methylation extraction). For both protocols, the BSBolt and gemBS pipelines also followed the corresponding alignment validation pipelines with each aligner adding the respective option to allow for non-directional alignment. The bwa-meth snmC-seq2 and Smallwood *et al.* pipelines followed the alignment validation pipeline for bwa-meth with no changes.

For Bismark, we followed the respective methods described in the publications for each method. snmC-seq2 and Smallwood *et al.* had slightly different alignment commands, but then followed a similar pipeline thereafter. Specifics of these pipelines can be found in the Supplemental Methods.

Read counts for BISCUIT, BSBolt, bwa-meth and gemBS were found in the same manner as the alignment validation. The Bismark results extracted the read names from the individual read BAMs, found the unique read names across both, then performed counting in the same manner as the alignment validation.

### Structural variant discovery

Sequencing data were downloaded from SRA (accession number SRR1800202) (33). The FASTQ files were processed with the biscuitSifter pipeline and then methylation extracted. Structural variants were called using manta (version 1.6.0) (34) and lumpy (version 0.2.13) (35). Manta was run in tumor-only analysis mode, with call regions determined by taking the inverse of the ENCODE hg38 exclusion list BED file, restricting to primary chromosomes, and removing the mitochondrial chromosome. Structural variants with lumpy were found using lumpyexpress.

### SNV validation and precision-recall curves

We used WGS data from Genome-in-a-Bottle (GIAB) and WGBS data (36) from the GM12878 cell line to validate single nucleotide variant (SNV) calling with BISCUIT. WGBS FASTQ files for two replicates were downloaded from SRA (SRA accession numbers SRR4235788 and SRR4235789) and trimmed using TrimGalore! (version 0.6.6 with cutadapt 4.1) and subsampled to 500 million reads each. The subsampled FASTQs were then aligned to hg38 and a pileup VCF created using BISCUIT. After the VCFs were created for each replicate, the intersection between the two was found, which was then filtered to remove SNVs with low genotype quality (GQ ≤ 5), that were not on the canonical chromosomes, or had a genotype of 0/0 relative to the reference. The resulting set of variants were used as the BISCUIT (i.e. WGBS) variants in the validation.

Two different datasets were used for GIAB. First, insertions and deletions were filtered from the high confidence variants VCF for NA12878 using vcftools (version 0.1.16) (37), leaving only the high confidence SNVs. These SNVs were used as the full GIAB set of variants during the validation process. Second, GIAB combines many types of sequencing technologies. Therefore, the Illumina-only FASTQ files were downloaded (the full list is available at github.com/genome-in-a-bottle/giab_data_indexes/blob/master/NA12878/sequence.index.NA12878_Illumina300X_wgs_09252015) to better compare with the Illumina-generated WGBS replicates. The individual FASTQ files were combined into a single file for reads 1 and 2 using pigz (version 2.4) and then downsampled to 500 million reads using seqtk. The downsampled FASTQ files were then aligned with BWA-MEM (version 0.7.17-r1188) (38) and duplicate marked with samblaster (version 0.1.26) (39). Variants were found using GATK (version 4.1.4.1). Germline variants were found and then SNVs were extracted and filtered based on GATK best practices. Additionally, variants not on canonical chromosomes or with a genotype of 0/0 relative to the reference were filtered to better match with BISCUIT. Note, GATK best practices has a stricter variant quality cutoff, so no additional filtering was applied for genotype quality. The variants that passed both sets of filters were used as the GATK (i.e. WGS) variants during validation. Once the three sets of variants had been found, the three-way intersection between the sets was found using bcftools.

To create the precision-recall curves, the BISCUIT and GIAB SNV data described above were restricted to the first 22 megabases of chromosome 11, phased haplotypes were converted to unphased, then intersected with the inverse of the ENCODE exclusion list and dbSNP (version 153) common SNPs. Precision and recall were calculated using the GIAB dataset as the ground truth. The curve labelled as ‘GQ ≥ *n*’ is drawn from the results as is, with no additional filtering applied. The curve labelled ‘GQ ≥ 15 (dbSNP+)/GQ ≥ *n* (dbSNP-)’ has an added filter where SNVs that intersect common dbSNP SNPs with a minor allele frequency (MAF) greater than 0.05 were allowed a GQ greater than or equal to 15, whereas all other variants were greater than or equal to *n*.

Based on the precision-recall curves, it was determined that a filter using a dbSNP prior would improve the false positive rate of BISCUIT SNV calling. To create the filtered BISCUIT SNVs, an additional filter was added to the previously described BISCUIT SNV calls. That filter retained SNVs that fell in the inverse of the ENCODE exclusion list and either intersected a common dbSNP SNP with a MAF ≥0.05 and genotype quality ≥15 or, if not, had a genotype quality ≥60. This newly filtered set of variants was then used for the final, filtered set of BISCUIT SNVs.

### EpiBED and allele-specific methylation

Whole-genome methylation profiling data from normal human fallopian tube samples from Morrison *et al.* (40) were used. We used two technical replicates each profiled with two methods (EM-seq with the New England Biolabs kit and WGBS with the Swift Biosciences kit). The data was aligned as described in (40), then each kit's technical replicates were merged, and individual epiBED files created for each kit. The resulting epiBED files were merged into a single epiBED file for generating the figure. The *SNRPN-SNURF* imprinted region was selected based on annotations for imprinted CpG probes in the Illumina MethylationEPIC array (41). After creating the epiBED file, it was imported into R using biscuiteer (version 1.13.1) and the figure was created using bisplotti (version 0.0.19, https://github.com/huishenlab/bisplotti). Reads were subsetted to those that covered both the SNP and CpG and sorted based on their methylation status.

## Results

### Overview of BISCUIT workflow and functionalities

A main goal when developing BISCUIT was to ensure the files output by the toolkit readily integrated with other tools. Therefore, BISCUIT natively produces standards-compliant file formats (e.g. SAM/BAM, VCF and BED) during the analysis process. Because of the standards-compliant philosophy, BISCUIT could be used to extract genetic and epigenetic information from SAM/BAM files aligned with other aligners (Supplementary Figure S2). Further, the SAM/BAM files produced by BISCUIT are compatible with the Integrated Genomics Viewer (IGV) (42) for visualizing DNA methylation, SNPs and structural variation. BISCUIT also provides its own utility for viewing SAM/BAM files that allows for simultaneous viewing of methylation and mutation status (Supplementary Figure S3).

**Figure 1.**
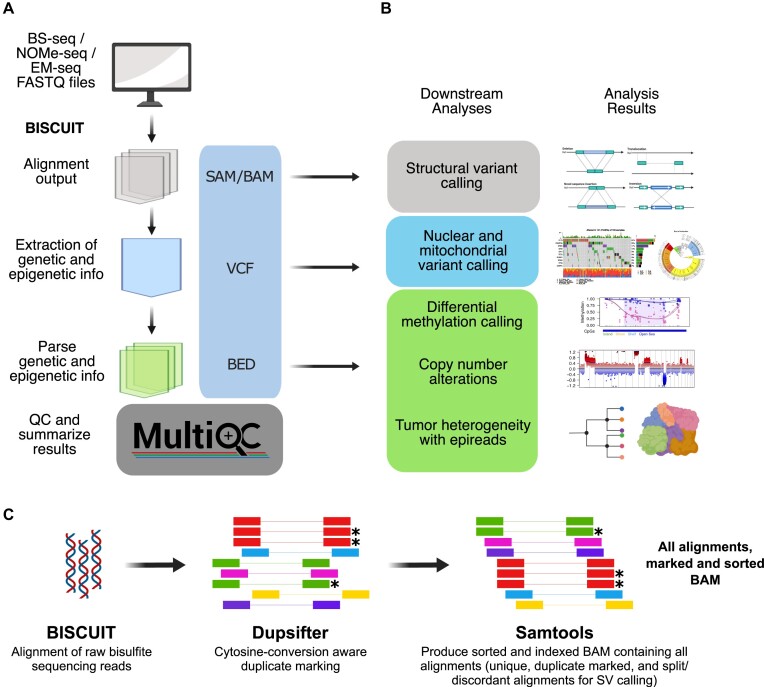
BISCUIT workflow produces standards-compliant file formats and readily integrates with downstream tools to infer genetic and epigenetic information. (**A**) BISCUIT workflow takes raw FASTQ files from WGBS or WGBS-like experiments and extracts genetic and epigenetic information through intermediate, standards-compliant file formats. (**B**) Intermediate analysis files can integrate with downstream tools that expect SAM/BAM, VCF and BED formatted inputs. (**C**) The combination of BISCUIT, dupsifter, and samtools enables rapid, accurate, and simultaneous DNA methylation read alignment, cytosine-conversion aware duplicate marking, and production of sorted and indexed BAMs (* represents a duplicate marked read).

Using thoughtfully designed bisulfite sequencing oriented quality control (QC) metrics, a summary HTML report can be generated using MultiQC (43) (Figure 1A). One QC metric example is the use of cytosine retention at non-CpG contexts for diagnosing cytosine conversion rates in different genomic regions and read positions. Whereas other tools monitor cytosine conversion through CpH (where H = A/C/T) retention (17), BISCUIT splits these CpH methylation contexts into CpA, CpC and CpT. By treating CpH methylation as a single unit, all CpHs are assumed to be unmethylated. However, CpH methylation has been observed in embryonic stem cells (44), neurons (45), and embryonal carcinomas (46), particularly at CpA sites. By comparing CpA versus CpC/CpT cytosine retention levels, the existence of non-canonical CpH methylation can be evaluated and the true background non-conversion rate inferred. BISCUIT also provides read filtering based on these non-conversion rates by excluding reads with extensive conversion failure, making it possible to salvage experiments where bisulfite conversion is less than optimal.

Given that genetic and epigenetic information intrinsically exist in WGBS data, BISCUIT extracts and aggregates these data into a VCF file that can be mined for locus-specific state information. Notably, BISCUIT can call somatic variants if both normal and disease BAMs are provided. Furthermore, NOMe-seq data are handled in a way that excludes cytosines at the GpCpG context while extracting accessibility and CpG methylation (Supplementary Figure S4). Base-level variation, whether SNVs or methylation states in a user-defined cytosine context, can then be summarized into a BED file. These intermediate file types can be leveraged for other downstream analyses beyond differential methylation calling (Figure 1B). As an example, the BAM output can be passed to tools like lumpy (35) or manta for large-scale structural variant calling. Another unique capability of BISCUIT is the integration of genetic and epigenetic states to yield single-molecule information in the epiBED format (described below), enabling analyses such as allele-specific methylation (47,48).

The key that unlocks much of the information potential in WGBS data on clinically relevant timescales is the coupling of BISCUIT’s alignment approach with dupsifter and samtools (Figure 1C). During alignment, BISCUIT pipes reads to dupsifter for duplicate marking then to samtools for sorting and indexing. This piping approach, called biscuitSifter, is part of what makes BISCUIT more efficient and scalable relative to existing tools (see Accuracy and Speed Benchmarking). Additionally, we have developed a portable Snakemake-based (49) workflow for creating analysis files from raw FASTQ files using the biscuitSifter approach and subsequent BISCUIT subcommands (Supplementary Figure S5). This workflow also incorporates the QC described above with additional bisulfite/enzymatic conversion diagnostics if spike-in control vectors, such as lamda phage or pUC19, are included in each sample (Supplementary Figure S6) (40).

### Indexing and alignment methodology

**Figure 2.**
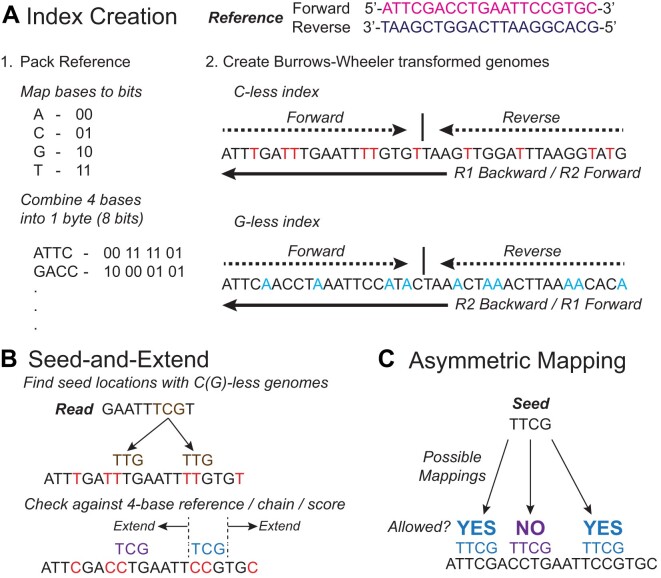
BISCUIT utilizes a packed reference and two 3-base Burrows-Wheeler transformed genomes with spaced FM-indexes, along with a seed-and-extend method, for efficiently and accurately mapping reads to the reference. (**A**) The reference FASTA is packed to reduce memory during alignment. Two Burrows-Wheeler transformed genomes with spaced FM-indexes are also created by making two copies of the forward and reverse strands concatenated together. One copy, the ‘C-less’ index, converts all C’s to T’s, while the other copy, the ‘G-less’ index, converts all G’s to A’s. (**B**) Seeds are generated by finding the longest string of bases that exactly map to one of the 3-base genomes. After the seeds have been generated and checked against the 4-base reference, they are chained together, extended, and scores are calculated. The highest scoring seed location is set as the mapping location. (**C**) BISCUIT utilizes an asymmetric scoring paradigm, where T’s (or A’s) in the read can map to C’s (or G’s) in the reference genome. However, the reverse is not allowed. Practically, this is handled by not penalizing C (reference) to T (read) or G to A mismatches when calculating the extension score.

BISCUIT uses a novel alignment approach built upon the Burrows-Wheeler aligner. The reference is indexed by creating a packed 4-base reference, as well as two 3-base Burrows-Wheeler transformed genomes with spaced Full-text indexes in Minute space (FM-indexes) (50). The two indices are both based on concatenations of the forward and the reverse strands, but one index is C-less and the other is G-less. The two indexed sequences are reverse-complementary to each other to allow a FM-index-based search in both directions (Figure 2A). Seed sequences (short sequences upon which alignments are created) are formed by *in silico* converting all C’s to T’s in a copy of the read and searching for exact matches of short portions of the read in the FM-indexes. Locations where exact matches occur are considered initial location candidates and are then filtered for compatibility with the 4-base reference, chained together by genomic proximity, and scored. The chain(s) with the highest score(s) are then chosen for seed extension, which is done against the 4-base reference using the original read, not against a 3-base converted reference with an *in silico* converted read. If perfect extension fails, a Smith-Waterman-like algorithm (51) is used to identify insertions and deletions (Figure 2B). During extension and Smith-Waterman alignment, the base substitution matrix allows conversion asymmetry (52), where a T (or A) can be aligned to a C (or G) in the genome, but not vice versa (Figure 2C). After mapping, BISCUIT reassesses the conversion strand for ambiguity. For highly repetitive regions, BISCUIT will only visit the seed chains with the highest scores (the number of which is set by the user), pending they all properly conform with conversion asymmetry. However, if too few valid seed chains can be identified, BISCUIT will keep visiting chains up to a user-defined maximum number of chains. In the case of equally optimal mapping, BISCUIT will report a mapping quality of 0. For details on the specifics of calculating the mapping quality, see the Supplementary Materials. If the number of alternative alignments (both equally optimal and suboptimal) is below a user-defined threshold, all alternative alignments will be output in the XA SAM auxiliary tag. Otherwise, the number of alternative alignments is output in the XB auxiliary tag.

### Single nucleotide polymorphism calling overview

There is uncertainty when calling SNPs from WGBS data due to the ambiguity of whether C to T or G to A conversions are due to the presence of a SNP or an unmethylated cytosine. BISCUIT uses a conservative strategy to calculate base support for SNPs. It does this by using Rs and Ys (from the International Union of Pure and Applied Chemistry (IUPAC) nucleotide codes) to represent the A’s and T’s seen in the G-less (original bottom/complement to the original bottom (OB/CTOB) strand) and C-less (original top/complement to the original top (OT/CTOT) strand) reads, respectively. This results in a base support alphabet that consists of six letters (A, C, G, T, R, Y). BISCUIT then tries to reduce this six-letter alphabet to the standard four-letter alphabet (A, C, G, T) by shifting the R and Y read support to the other four letters. The process to do this (shown for Y, but redistributing R is similar in principle) is as follows:

- When unambiguous evidence (either from the OB/CTOB strand or by observing an unconverted base) supporting the presence of *one* base (C or T) is present, BISCUIT adds the Y allele support to the support of that corresponding base.
- When unambiguous evidence supporting C *and* T is seen, BISCUIT ignores the Y allele support (it is not added to either base).
- When unambiguous evidence is missing for both C and T and the reference is not a C or T, then the Y allele support is also ignored.
- When unambiguous evidence is missing for both C and T and the reference is either a C or a T, then the Y allele support is added to the reference allele support.

In other words, BISCUIT infers the allele support when unambiguous evidence supports one base or the other, or when the reference could explain the ambiguity if direct evidence is not present. After reducing the allele support to a four-base alphabet, BISCUIT determines the genotype and somatic mutation status using a Bayesian model parameterized by the contamination rate, sequencing error rate, and empirical polymorphism rate (53).

### Comparison to other aligners

Several cytosine-conversion-aware aligners already exist, from the widely used Bismark and bwa-meth to the more recent BSBolt and gemBS. While there are many similarities among these tools (Table 1), there are some key differences that distinguish BISCUIT from the others. While BISCUIT, Bismark, BSBolt and bwa-meth all generate 3-base FM-indexes, only BISCUIT checks for 4-base reference compatibility early in alignment and scores the mapping candidates against the full 4-base reference seeds (gemBS uses a different alignment methodology, although it performs 3-base alignments like Bismark, BSBolt and bwa-meth, and will not be included in this discussion). On the other hand, Bismark, BSBolt, and bwa-meth align and score against a 3-base reference, whether that is using a single index with a single concatenated reference (BSBolt and bwa-meth) or one index for each conversion (Bismark). On a related note, only BISCUIT allows for conversion asymmetry, while the other three do not. By allowing for this asymmetry, BISCUIT behaves in a manner that is closer to reality over aligning to a 3-base reference. While Bismark and BSBolt also make use of a 4-base reference during alignment to assess methylation, BISCUIT uses the 4-base reference both to assess methylation and to score alignments.

**Table 1.** Comparison of BISCUIT, Bismark, BSBolt, bwa-meth and gemBS. Tools in parentheses are third party tools used to perform the stated functionality

	BISCUIT	Bismark	BSBolt	bwa-meth	gemBS
**General**					
Language	C	Perl	Python/C	Python/C	Rust/C
Multi-threaded	Yes	Yes	Yes	Yes	Yes
Availability	GitHub, Bioconda, Docker	GitHub, Bioconda	GitHub, Bioconda, Pip	GitHub, Bioconda	GitHub, Bioconda, Docker
**Supported Libraries (WGBS, EM-seq, RRBS, PBAT, NOME-seq)**					
Mode	Directional, Non-Directional	Directional, Non-Directional	Directional, Non-Directional	Directional	Directional, Non-Directional
**Index / Algorithm**					
Reference	4-base	3-base	3-base	3-base	3-base
Seed Creation	3-base	3-base	3-base	3-base	3-base
Asymmetric Scoring	Yes	No	No	No	No
Alignment Algorithm	BWA-MEM-based	Bowtie2 / HiSAT2	BWA-MEM-based	BWA-MEM	GEM3
Global or Local Alignment?	Local	Global	Local	Local	Both
**Functionality**					
Handle Spike-ins	Yes	Yes	Yes	Yes	Yes
UMI Support	Yes	Yes	No	No	No
Handle Cell Barcodes	Yes	Yes	No	No	No
5′/3′ Trimming	Yes	(TrimGalore!)	(TrimGalore!)	(TrimGalore!)	BScall
Duplicate Marking	(dupsifter)	Yes	(samtools)	(Picard)	BScall
Collapse Overlapping PE Reads	Yes	Yes	Yes	No	BScall
Variant Calling	Yes	No	Yes	(BISCUIT)	Yes
Methylation Extraction	Yes	Yes	Yes	(MethylDackel)	Yes
Accessibility Extraction	Yes	Yes	No	No	No
Companion R Tool	Yes	Yes	No	No	No
Visualize WGBS BAM	Yes	No	No	No	No
**Input / Output**					
FASTA / FASTQ	Yes	Yes	Yes	Yes	Yes
Standard Input	Yes	No	No	Yes	Yes
Write Directly to SAM/BAM	No	Yes	Yes	No	Yes
Write directly to Standard Output	Yes	No	Yes	Yes	No
CpG and CpH	Yes	Yes	Yes	No	Yes
**Quality Control**					
MultiQC Support?	Yes	Yes	No	No	No
Non-CpG Stats	CpA / CpC / CpT	CpH	CpH	No	No
M-Bias Plot	CpG / CpH	CpG / CpH	No	No	CpH

In terms of the alignment methodology, BISCUIT, BSBolt and bwa-meth are built on the BWA-MEM algorithm, Bismark uses Bowtie2, and gemBS uses the GEM3 aligner. Of these five tools, Bismark, bwa-meth and gemBS all serve as wrappers around their respective alignment algorithms, whereas BISCUIT and BSBolt started from the BWA-MEM algorithm and modified it to account for differences between WGS and WGBS. Because they are based on BWA-MEM, BISCUIT, BSBolt, and bwa-meth use local alignment, versus Bismark's global (or end-to-end) alignment. Due to the difference in GEM3’s alignment strategy, gemBS is able to perform both local and global alignments.

The output from Bismark, BSBolt (by default) and gemBS are written straight to a SAM/BAM file, while the output from BISCUIT and bwa-meth (and by user specification from BSBolt) are sent to the computer's standard output data stream. By streaming the output, alignment, duplicate marking, and coordinate sorting can be combined into a single step, rather than individual steps for each (as is generally done during mapping in gemBS).

All five aligners are able to map NOMe-seq data; however, only BISCUIT and Bismark include the option to extract methylation related to accessibility. When performing NOMe-seq, methylation due to off-target activity of the M.CviPI enzyme in endogenous CpCpG contexts has been seen (6). The off-target methylation is a small effect (<5%); therefore, BISCUIT does not filter out methylation occurring in a CCG context. Bismark, on the other hand, does filter out these methylation contexts. Rather than removing half of all possible cytosines from the analysis pool for a small off-target effect, BISCUIT includes the CpCpG context methylation and allows the user the choice to filter these post-hoc in analyses where this effect may be a problem.

To date, Bismark has been used most frequently in single-cell analyses; therefore, it has some capabilities to handle single-cell-specific items, such as cell barcodes and unique molecular indexes (UMIs). However, it must rely on third-party tools to perform much of the preparation of single-cell FASTQs for input into Bismark. BISCUIT, on the other hand, is able to extract barcodes, with the output able to be piped straight into BISCUIT for alignment. In addition, if cell barcode correction must be performed, BISCUIT is compatible with FASTQs processed by UMI-tools (54) for cell barcode correction and extraction.

### Accuracy and speed benchmarking

**Figure 3.**
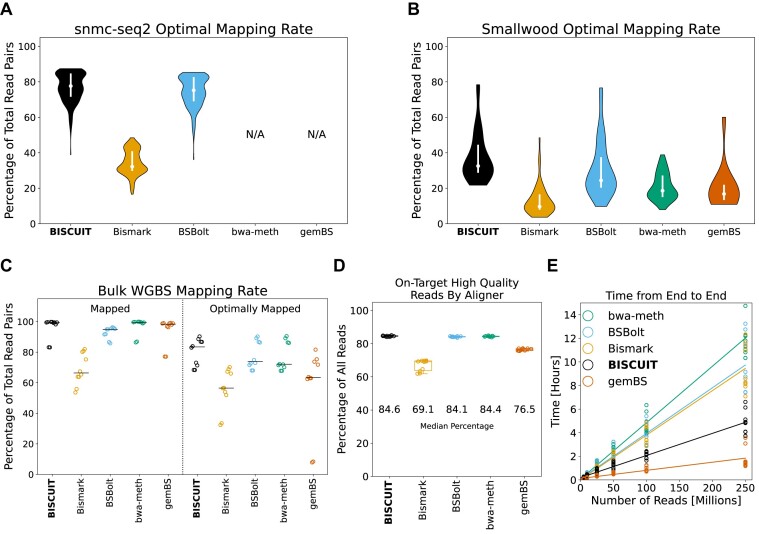
Alignment recovery, accuracy, and speed benchmarking for single-cell and bulk WGBS datasets. (**A**) Optimal mapping rate by BISCUIT, Bismark, BSBolt, bwa-meth and gemBS for snmC-seq2 data. bwa-meth and gemBS were unable to align FASTQs from the snmC-seq2 data. (**B**) Same as (A), but for Smallwood *et al.* data. (**C**) Percentage of 250 million bulk WGBS reads mapped and optimally mapped for the five aligners. ‘Mapped’ reads include only primary alignments. ‘Optimally mapped’ reads include primary alignments with a minimum mapping quality score (MAPQ) of 40. (**D**) Fractional distribution of all TruSeq Methyl Capture EPIC reads that were on-target and optimally mapped for each aligner. (**E**) The time spent to go from FASTQs to extracted methylation for varying numbers of reads for bulk WGBS alignment.

For single-cell WGBS samples, we compared five aligners (BISCUIT, Bismark, BSBolt, bwa-meth and gemBS) on previously generated data from two different protocols, snmC-seq2 (30) and Smallwood *et al.* (31). For these single-cell datasets, the recommended alignment pipeline uses Bismark and aligns reads 1 and 2 from paired-end sequencing separately, likely due in part to chimeric reads produced during the linear amplification step of these protocols (55). However, this breaks up the correlated nature of paired-end sequencing for non-chimeric reads and decreases the ability to adequately account for the PCR duplicate rate across all reads, increasing analysis complexity. By not explicitly requiring alignment of whole reads, BISCUIT (and likewise BSBolt) can align both chimeric and non-chimeric reads simultaneously, retaining the paired-end nature of the data in non-directional mode, decreasing analysis complexity. Paired with the biscuitSifter pipeline, this enables rapid scaling to large single-cell WGBS experiments. When comparing the rate of optimally mapped reads for both datasets (Figure 3A, B), BISCUIT outperforms the other aligners (although BSBolt performs nearly as well on the snmC-seq2 data). For both datasets, Bismark was run in both single-end mode and paired-end mode. However, the mapping rates were so poor (<5% on average) in paired-end mode that they were excluded from Figure 3. Several attempts were made to recover some level of optimal alignments by adjusting alignment parameters in the command line invocation, but all resulted in similar levels of optimal alignment. Both bwa-meth and gemBS failed in aligning snmC-seq2 data, likely due to empty or very short (<20 bp in length) reads in the FASTQs, which both aligners are unable to handle. Additionally, for gemBS, only a subset of the Smallwood *et al.* data was processed (30 out of 49 samples, see Supplementary Table S3 for which samples). When trying to align these FASTQs, gemBS was unable to finish aligning 18 of the 30 attempted samples in less than two days of running time. Because the other four aligners were able to finish aligning all datasets in less than a day, the decision was made to not process the remaining 19 samples. Therefore, only the 12 samples that completed alignment in less than two days are shown in Figure 3B.

For bulk WGBS samples, Bismark aligned a substantially lower number of total and optimally aligned reads compared to the other aligners (Figure 3C; Supplementary Figure S7). While the other four aligners had a comparable fraction of total mapped reads, BISCUIT had a higher median fraction of optimally aligned reads. To assess alignment accuracy in bulk WGBS, we used ten publicly available datasets generated with the Illumina TruSeq Methyl Capture EPIC library preparation kit, which targets a set of known regions. BISCUIT, BSBolt and bwa-meth, which are all based on BWA-MEM, had the highest median fraction of reads that were on-target and optimally mapped, with BISCUIT having a slightly higher fraction than the other two aligners (Figure 3D).

Of the three BWA-MEM based aligners, BISCUIT was the fastest going from raw FASTQs to extracted methylation levels (Figure 3E; Supplementary Figure S8). It was only slower than gemBS, which had lower accuracy in the capture sequencing benchmarking (Figure 3D).

### WGS-like structural variation and SNP analyses

**Figure 4.**
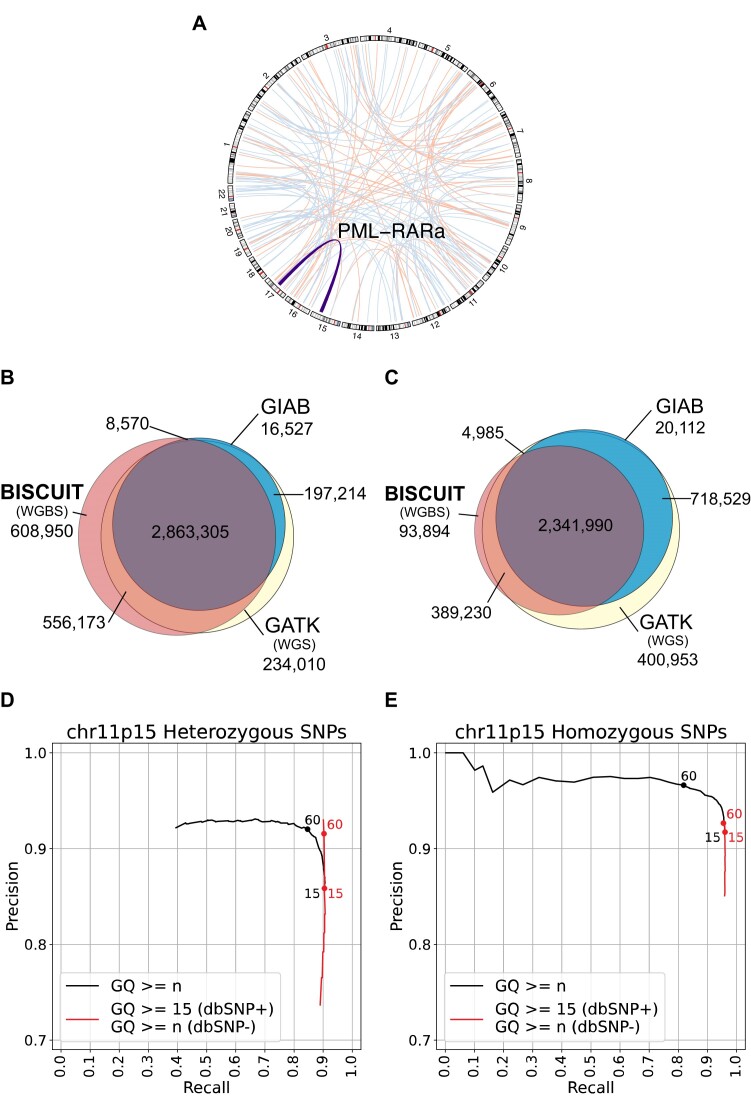
Leveraging its use of standards-compliant file formats, BISCUIT can be used to find SNPs and SVs from WGBS data. (**A**) Data aligned with BISCUIT identifies large-scale structural variants, including the clinically relevant PML-RARα translocation, with both manta (red) and lumpy (blue). (**B**) The intersection of SNPs in GM12878 found by BISCUIT (using WGBS data), GATK (using Illumina-only Genome-in-a-Bottle (GIAB) WGS data) and GIAB joint variant calls. (**C**) After applying genotype quality filtering based on (D) and (E), the fraction of SNPs unique to BISCUIT drastically decreases. (**D**) Precision-recall curve of heterozygous SNPs on chromosome 11p15. ‘GQ ≥ *n*’ filters strictly based on the genotype quality score (GQ), while ‘GQ ≥ 15 (dbSNP+) / GQ ≥ *n* (dbSNP-)’ filters by GQ ≥ 15 for dbSNP common alleles and GQ ≥ *n* otherwise. (**E**) Precision-recall curve for homozygous SNPs on chromosome 11p15. Filters are applied in the same way as (D).

As a demonstration of BISCUIT’s ability to readily integrate with existing tools to extract large SV events from DNA methylation sequencing data, we reanalyzed methyl capture bisulfite-sequencing data from the AML Sequencing Project (33). Using BISCUIT output, manta and lumpy identified a list of translocations, including the clinically relevant PML-RARα translocation (Figure 4A). To show BISCUIT recovers SNPs like WGS, we reanalyzed two GM12878 WGBS datasets and compared them against Genome-in-a-Bottle (GIAB) and reanalyzed Illumina-only WGS datasets from GIAB. Without stringent filtering, BISCUIT recovers 93% of SNPs in the GIAB ‘truth’ set and almost 89% of SNPs found in WGS (Figure 4B). We also explored the precision-recall of BISCUIT SNPs across genotype quality (GQ) thresholds on chromosome 11p15 (Figure 4D–E). By filtering SNPs with GQ ≥ 15 that overlap common SNPs found in dbSNP and GQ ≥ 60 otherwise, the false positive rate decreased from 14% to 8% for heterozygous SNPs (Figure 4D). Applying this filtering to the intersection of SNPs between WGBS, WGS, and GIAB reduces the fraction of SNPs unique to BISCUIT from 15% to 3% (Figure 4C). Taken together, BISCUIT can extract diverse forms of genetic information from WGBS with high fidelity.

### epiBED: extending the epiread and epiallele formats

**Figure 5.**
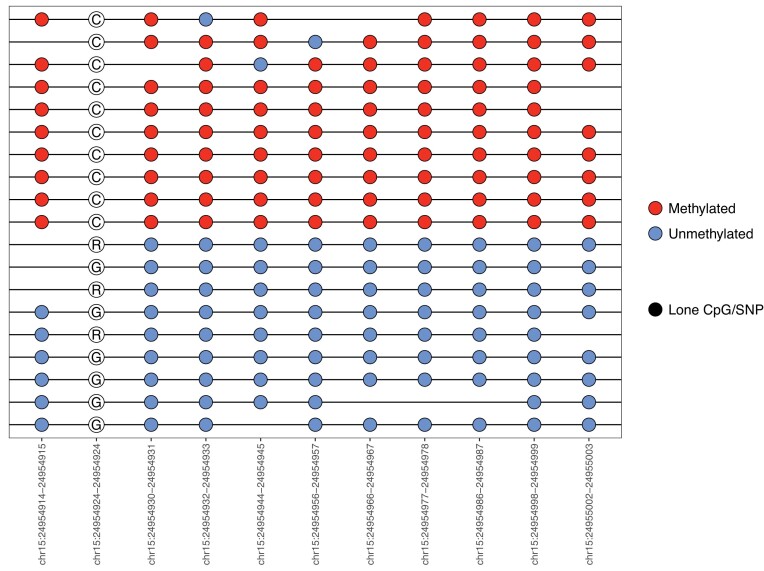
The epiBED format can be used to find allele-specific methylation in the canonical imprinted *SNRPN-SNURF* region. Ambiguous bases (‘R’) in the SNP are due to the collision of G to A SNPs versus the G to A conversion in bisulfite sequencing. When this occurs, BISCUIT labels the base as ‘R’ (or ‘Y’ in the case of C to T conversions). Biscuiteer and bisplotti do not redistribute any ambiguous base calls, but it is possible to redistribute bases based on the reference base and the distribution of bases at that locus. In this instance, the R’s can be understood to be G’s.

The *epiread* and *epiallele* formats provide a compact way to represent read-level and single-molecule methylation that can be used to facilitate the study of intra-tumoral heterogeneity (48,56). BISCUIT incorporates a modified *epiread* format which contains SNP information, making it the first of such to co-store epigenetic and SNP information. However, these two formats require external CpG and SNP coordinates as references and cannot be easily converted to BED files for region-specific epi-haplotype rendering. Neither can they be easily turned into a matrix-like format for calculating co-occurrence-based information metrics and visualization. To address these limitations, BISCUIT expands the *epiread* and *epiallele* formats to a new, unified format, called epiBED. It is BED-compliant and captures genetic and epigenetic information through read-level run-length encoding (RLE) (Supplementary Table S4). When encoding the per-read base-level information, BISCUIT can adaptively filter low-quality bases and is mate overlap-aware to prevent double counting of redundant methylation derived from the same molecule. EpiBED can also be readily converted to WIG and bigWig formats. Using biscuiteer, DNA methylation from mate reads can be combined to form a physically ‘phased’ epi-haplotype, allowing for single-molecule level analysis. Further, representing the data in a BED-compliant fashion enables efficient compression and indexing through tools like bgzip and tabix that downstream tools can use to rapidly extract regions of interest for further analysis. While BISCUIT’s epiBED format is a transparent, data-rich view of per-read methylation and mutation status, the prior *epiread* and *epiallele* formats are retained as optional output for existing tools that expect these formats as input (57,58). To show the utility of the epiBED format to find allele-specific methylation, the canonical imprinted region, *SNRPN-SNURF*, was used (Figure 5). Two distinct methylation states (one almost entirely methylated, the other unmethylated) can be seen, with a G to C SNP corresponding to each state (G in the unmethylated allele, C in the methylated allele). It should be noted that while finding allele-specific methylation is feasible, it is a difficult task. With sufficient depth, it can be easy to find CpGs and SNPs that have correlated methylation and base states. However, many such cases occur when a SNP directly alters the CpG, which BISCUIT can resolve (Supplementary Figure S9).

### Biscuiteer: integrating BISCUIT output with the R/bioconductor analysis ecosystem

We introduce the R/Bioconductor package, biscuiteer, which converts BISCUIT VCF, BED and epiBED output into standard Bioconductor structures, permits out-of-core analysis of large experiments, and integrates with the full complement of Bioconductor analysis packages (Supplementary Figure S10). Analysis of WGBS datasets from even a few samples may exceed the memory limits of most laptops and desktop workstations. To facilitate the downstream exploratory analysis of base-resolution bulk and single-cell methylomes, biscuiteer reads BISCUIT output into the bsseq (59) data structure, which can be HDF5-backed to support out-of-core computation and passed to various downstream tools, (e.g. dmrseq (60)) that support bsseq-like data structures in R. Copy number variations can be detected from WGBS data, either directly from the BAMs or by using the coverage across all cytosines to provide a copy number ‘sketch’ (61). Biscuiteer further integrates with existing CNV annotation tools, allowing users to link structural variation with epigenetic variation (62,63). Allele-specific methylation can be found using the epiBED format, which biscuiteer converts into a read-level or single-molecule-level GRanges object of methylation, accessibility (if using NOMe-seq data), and SNP states. Biscuiteer is a flexible, memory-conscious interface between BISCUIT output and the R/Bioconductor ecosystem of analysis tools.

## Discussion

With the growing popularity of multi-omics methods for dissecting underlying molecular mechanisms driving observed phenotypes in normal and diseased tissues, we highlight the utility of WGBS approaches, as they inherently possess multi-omic data types on multiple levels: on the same read, from the same sample, and in a single assay. Thus, we developed an end-to-end toolchain, BISCUIT and biscuiteer, that can rapidly and accurately process WGBS data to maximally extract genetic and epigenetic information from DNA methylation sequencing experiments.

In this work, we demonstrated its utility to recover SVs and SNVs from WGBS and WGBS-like experiments. Another group showed that BISCUIT performed better than bwa-meth and other existing methods for indels, despite BISCUIT not being specifically designed for indel handling (64). An additional independent study validated BISCUIT’s performance for SNP calling in *Parus major* (65), albeit with an earlier version of BISCUIT. Of all the tools benchmarked by the group, BISCUIT had the highest sensitivity. In this manuscript, we showed how to properly filter the SNP calls for maximized sensitivity and specificity. BISCUIT enables the cost-effective joint characterization of genetic variation (from point mutations to complex structural variants) and epigenetic variation (DNA methylation via WGBS and phased chromatin accessibility via NOMe-seq). The results directly link genetic and epigenetic alterations on individual molecules to permit fast, powerful analysis of biological processes across multiple loci, even in template-scarce experiments.

BISCUIT produces intermediate, standards-compliant file types that can readily integrate with existing bioinformatics software tools to aid in specialized downstream analyses, such as structural variant or differentially methylated region detection. Given the high number of WGBS datasets that already exist due to large sequencing initiatives such as ENCODE, TCGA and ROADMAP, in conjunction with the increasing amount of EM-seq, cell-free BS-seq and single-cell WGBS data and approaches, we expect BISCUIT and biscuiteer to provide broadly applicable methods to readily analyze legacy and future WGBS data in research and translational settings.

Tool comparison is a difficult process, and bottlenecks often exist in pipelines which can vary across different computational infrastructures. We attempted to adhere as closely as possible to best practices for processing data with each tool, but there may be other factors that we have not considered. With that in mind, BISCUIT can go from raw FASTQs to extracted methylation levels in less time when compared to Bismark, BSBolt, and bwa-meth. In this paper, we presented speed benchmarks, but BISCUIT also has minimal demand on memory compared to existing tools, particularly for hard disk space. It uses less disk space for the references index, as it does not need to store any 3-base references or additional tool-specific index files. Further, it requires fewer intermediate files than the other aligners when generating methylation BED files from raw FASTQs. This also makes BISCUIT particularly suited for large-scale studies.

Long-read sequencing methodologies, such as the various Pacific Biosciences and Oxford Nanopore Technology platforms, have emerged as novel approaches for global DNA methylation profiling, excelling at resolving epi-haplotypes across extended genomic DNA stretches. These approaches can profile epigenetic modifications utilizing either direct detection (66) or decoding amplified base-converted sequences (67,68). While we discussed BISCUIT largely for short-read sequencing, BISCUIT is compatible with long-read base-converted experiments, such as LR-EM-seq (68). In addition, BISCUIT’s epiBED format allows for a compact, read-level representation of epigenetic modification and genetic variation information, thereby offering an opportunity to investigate selective cytogenetic force on specific epigenetic and/or genetic/epigenetic patterns in CpG-sparse regions, repetitive regions, and regions with amplification bias. The epiBED format can serve as an infrastructure for long-range methylome phasing (69) and for studying allele-specific epigenetic regulation such as at imprinting sites (70).

DNA cytosine modifications often display spatial autocorrelation due to enzyme processivity (71). As a result, read-level information from short-read sequencing data can provide mutually correcting evidence and protection against sequencing errors and stochastic epigenetic drift. Coordinated differential methylation at the block level offers more sensitive and robust indicators of distinct cellular identities (72) and malignancy in cell-free DNA (73,74). The epiBED format enables efficient filtering and extraction of read-level data into a matrix format and hence facilitates the analysis of information entropy and other heterogeneity measures of read-level methylation discordance (56,74).

Furthermore, we showed that single-cell DNA methylation data analysis can greatly benefit from BISCUIT’s higher mapping sensitivity and base-mismatch tolerance. BISCUIT’s position-independent seed-and-extend strategy can align chimeric inserts formed from hairpin ligation or linear pre-amplification before adapter tagging. The BISCUIT epiBED format can also be used to compactly store sparse single-cell methylome data, as single-cell methylomes are allelic and can be digitized assuming fully methylated, unmethylated, or mono-allelically methylated states (75). BISCUIT’s flexibility and ability to analyze compact read-level methylome data make it an indispensable asset to help researchers to reveal the complex interplay between genetic and epigenetic factors in health and disease.

Besides bisulfite conversion, BISCUIT offers integrated computational solutions for analyzing other epigenomic profiling data derived from cytosine conversion principles, such as NOMe-seq (6), TAB-seq (76), ACE-seq (77), TAPS (5) and hairpin-based techniques like five- and six-letter sequencing (78). These methods may have decoding rules based on sequence contexts (CpG versus non-CpG), the chemicals or enzymes employed for conversion, or the read's position on the insert. For instance, BISCUIT inherently supports decoding genetic variation from BS-seq, chromatin accessibility from NOMe-seq, 5-hydroxymethylcytosine from TAB-seq and ACE-seq and 5-methylcytosine from TAPS. It can be easily adapted to interpret genetic variation and cytosine modifications from hairpin-based sequencing approaches. The versatile encoding of multiple epigenetic and genetic information by BISCUIT allows the study of ‘read-level multi-omics’ such as would be revealed by methods like NOMe-seq.

In summary, while other cytosine-conversion-aware aligners have their merits, we believe BISCUIT represents a comprehensive and powerful tool. For both single-cell and bulk experiments, BISCUIT outperforms other aligners in the fraction of optimally aligned reads. BWA-MEM-based aligners generally produce higher alignment accuracy. Of these aligners, BISCUIT goes from raw FASTQ files to extracted methylation faster. Qualitatively, the allowance of conversion asymmetry (and the related scoring scheme) better reflect the reality of cytosine conversion. By streaming aligned reads to the standard output data stream, BISCUIT can readily combine with other tools for duplicate marking and coordinate sorting, allowing for easier scalability to large experimental setups. Further, GpC methylation levels for genomic accessiblity in NOMe-seq can be extracted with BISCUIT for all non-ambiguous cytosine contexts. In addition to processing bulk WGBS datasets, BISCUIT provides a substantial improvement for the rate of optimally aligned reads from single-cell WGBS experiments when compared with Bismark, the most popular aligner used for single-cell WGBS. BISCUIT also includes cell barcode extraction capabilities and seamlessly works with UMI-tools for cell barcode correction. The companion R package, biscuiteer, allows for easy input of methylation levels and epiBED files to R for use in existing analysis tools. BISCUIT’s speed, efficiency, and wide utility across many experimental setups makes it the perfect tool for analyzing methylation-related datasets.

## Supplementary Material

gkae097_Supplemental_File

## Data Availability

The data underlying this article are listed in the article and in the online Supplementary material. BISCUIT source code for versions used in this analysis can be found on Zenodo at the following DOIs: https://doi.org/10.5281/zenodo.10480760 (version 1.1.0) and https://doi.org/10.5281/zenodo.10480900 (version 1.2.1).

## Code availability

BISCUIT source code: https://github.com/huishenlab/biscuit

BISCUIT documentation: https://huishenlab.github.io/biscuit/

BISCUIT Snakemake pipeline: https://github.com/huishenlab/Biscuit_Snakemake_Workflow

Biscuiteer: https://www.bioconductor.org/packages/release/bioc/html/biscuiteer.html

Development version of biscuiteer: https://github.com/trichelab/biscuiteer

## Notes

Present address: Wanding Zhou, Center for Computational and Genomic Medicine, Children's Hospital of Philadelphia, Philadelphia, PA 19104, USA.
