# Supplementary material for: BISCUIT: an efficient, standards-compliant tool suite for simultaneous genetic and epigenetic inference in bulk and single-cell studies
Source: Nucleic Acids Res. 2024 Feb 27;52(6):e32. doi: 10.1093/nar/gkae097 (PMC11014253; doi:10.1093/nar/gkae097)
Supplement: gkae097_Supplemental_File [file gkae097_supplemental_file.pdf]

BISCUIT Supplemental Materials

BISCUIT Authors

Contents

1 **Calculating BISCUIT Mapping Quality Scores** **1**
1.1 Overview 1
1.2 User-Defined Parameters and Precalculated Values 2
1.3 Mathematical Description of Calculation 3
1.3.1 Mapping Quality for Single-End Alignment 3
1.3.2 Calculating Insert Size Statistics 4
1.3.3 Mapping Quality for Paired-End Alignment 5

2 **Supplementary Methods** **7**
2.1 Alignment Validation 7
2.2 Speed Benchmarking 12
2.3 Single-cell WGBS Alignments 13
2.4 Structural Variant Discovery 16
2.5 SNV Validation and Precision-Recall Curves 17
2.6 EpiBED and Allele-Specific Methylation 19

3 **Supplementary Tables** **20**

4 **Supplementary Figures** **22**

References **32**

1 Calculating BISCUIT Mapping Quality Scores

1.1 Overview

According to the SAM file specification[1], the mapping quality score[2] is the Phred score for the probability the mapping position is wrong ("$-10\ln(10)\Pr\{\text{mapping position is wrong}\}$, rounded to the nearest integer"). There are three primary sources of error related to mapping[3]:

1. the read does not come from the reference aligned to,

[1]`https://samtools.github.io/hts-specs/SAMv1.pdf`

[2]The mapping quality score is typically styled as "MAPQ," but here will be referred to as Q_{map} throughout. This is done merely to ease notation.

[3]From the MAQ Supplementary materials.

2. the true position is missed by the aligner, and

3. the hit is not correct.

Practically, the mapping quality score calculated by BISCUIT is dependent on parameters used in the Smith-Waterman (SW) algorithm, the calculated SW score, the number of sub-optimal alignments, the fraction of the alignment covered by seeds (short fragments that perfectly match the reference), and the repetitiveness of the seeds. Further, the length of the alignment region and whether the read is single-end or paired-end affects the mapping quality score. Here, we provide the methodology behind the mapping quality score in BISCUIT. Note, all values provided throughout are for BISCUIT version 1.2.1.

1.2 User-Defined Parameters and Precalculated Values

The mapping quality score is based on several elements that are controlled through the command line interface of `biscuit align` and values calculated prior to determining the mapping quality score. The tables below show the values that are controlled through the command line and the values that are calculated ahead of time.

Variable	Definition	Command Line Argument	Default Value
A	Score for match in SW algorithm	`-A INT`	1
B	Penalty for mismatch in SW algorithm	`-B INT`	2
C	Mapping quality coefficient length	`-Q INT`	50[4]
D_o/I_o	Gap-open penalty for a deletion or insertion	`-O INT[,INT]`	6,6
D_e/I_e	Gap-extension penalty for a deletion or insertion	`-E INT[,INT]`	1,1
L	Minimum seed length	`-k INT`	19
U	Penalty for unpaired read pair	`-U INT`	17

Command Line Parameters

Variable	Definition
F	Fraction of read covered by seeds
ϕ	Fraction of read covered by repetitive seeds
N	Approximate number of sub-optimal alignments
$\mathcal{S}$	Best SW score
s_{tandem}	SW score for hit in a tandem repeat (may or may not be calculated)
s_{sub}	Second best SW score (may or may not be calculated)

Precalculated Values

[4] Not documented, so value is fixed unless otherwise known.

1.3 Mathematical Description of Calculation

1.3.1 Mapping Quality for Single-End Alignment

First, we define the length of the alignment, λ, as the longer of the query (read) length or the reference length spanned by the alignment.

$$\lambda = \begin{cases} q_e - q_b & \text{if } q_e - q_b > r_e - r_b \\ r_e - r_b & \text{otherwise.} \end{cases} \tag{1}$$

Here, q_b and q_e are the beginning and end positions of the query sequence in the alignment, respectively. r_b and r_e are the corresponding beginning and end positions of the reference sequence in the alignment.

Next, we need to determine the second best Smith-Waterman score, s.

$$s = \begin{cases} s_{tandem} & \text{if } s_{tandem} \text{ set} \\ s_{sub} & \text{if } s_{tandem} \text{ is not set, but } s_{sub} \text{ is} \\ L/A & \text{otherwise,} \end{cases} \tag{2}$$

where s_{tandem}, s_{sub}, L, and A are defined in Section 1.2. If s is at least as good as $\mathcal{S}$ (i.e., $s \geq \mathcal{S}$), then the mapping quality score, Q_{map} is set to 0 and no further calculation is performed.

If $s < \mathcal{S}$, we next calculate two factors used in calculating Q_{map}. The first is a scaling factor, α, based on the difference between a perfect match SW score and the calculated top SW score.

$$\alpha = 1.0 - \frac{\lambda A - \mathcal{S}}{\lambda(A+B)}, \tag{3}$$

where λ is defined by Equation 1 and A, B, and $\mathcal{S}$ are defined in Section 1.2. The other value is used in Case 2 for calculating Q_{map} (see below).

$$\tau = \begin{cases} \alpha^2 & \text{if } \lambda < C \\ \alpha^2 \times \ln(C) \ / \ \ln(\lambda) & \text{otherwise} \end{cases} \tag{4}$$

There are now 3 possible ways to calculate an initial mapping quality score, $q_{initial}$:

1. If $\mathcal{S} = 0$, then

$$q_{initial} = 0. \tag{5}$$

2. If $\mathcal{S} > 0$ and $C > 0$, then

$$q_{initial} = \left\lfloor \frac{6.02 \times (\mathcal{S} - s) \times \tau^2}{A} + 0.499 \right\rfloor, \tag{6}$$

where 6.02 is a factor that includes both the conversion to the Phred scale and a constant needed for the approximation of the error due to the repetitiveness of the genome[5]. Adding 0.499 and taking the floor of the equation accounts for rounding $q_{initial}$ to the nearest integer (used regularly throughout).

[5]See the MAQ Supplementary materials for more details.

3. If $\mathcal{S} > 0$ and $C = 0$, then

$$q_{temp} = \left\lfloor 30.0 \times \left(1.0 - \frac{s}{\mathcal{S}}\right) \times \ln(F) + 0.499 \right\rfloor \tag{7}$$

$$q_{initial} = \begin{cases} \lfloor \alpha^2 \times q_{temp} + 0.499 \rfloor & \text{if } \alpha < 0.95 \\ q_{temp} & \text{otherwise,} \end{cases} \tag{8}$$

where 30.0 is a hard-coded coefficient and F is described in Section 1.2.

Once $q_{initial}$ has been defined, the final mapping quality score is calculated by adjusting for any sub-optimal alignments and any repetitive seeds used in generating the alignment.

1. Adjust for sub-optimal alignments:

$$Q_{map} = \begin{cases} q_{initial} - \lfloor 4.343 \times \ln(N + 1) + 0.499 \rfloor & \text{if } N > 0 \\ q_{initial} & \text{otherwise} \end{cases} \tag{9}$$

where N is defined Section 1.2 and 4.343 is needed to convert to the Phred scale.

2. Restrict Q_{map} to the fully closed boundary [0, 60]:

$$Q_{map} = \begin{cases} 60 & \text{if } Q_{map} > 60 \\ 0 & \text{if } Q_{map} < 0 \\ Q_{map} & \text{otherwise.} \end{cases} \tag{10}$$

3. Adjust by the fraction of the alignment covered by repetitive seeds:

$$Q_{map} = \lfloor Q_{map} \times (1.0 - \phi) + 0.499 \rfloor, \tag{11}$$

where ϕ is defined in Section 1.2.

Equation 11 is the mapping quality score used for individual reads, whether those are from single-end alignments or paired-end alignments with no primary alignments in one of the mates or BISCUIT is run with the `-P` option to skip pairing.

1.3.2 Calculating Insert Size Statistics

If the alignment is being performed on paired-end reads, then statistics must be calculated that govern valid insert sizes for properly paired reads. For a set of reads on a given processing thread, a list of approximate insert sizes are generated. Using the first two possible alignments for a read pair, the mates are checked to ensure both reads have at least one possible mapping, they both fall on the same bisulfite strand[6] and chromsome, and the score for the first redundant alignment[7] is sufficiently smaller than the best alignment (defined as $s_{redundant} \leq 0.8 \times \mathcal{S}$). For those reads that meet these criteria, the insert size, $\mathcal{I}$, is calculated as the

[6]The bisulfite strand can either be from the original top / complement to the original top (OT/CTOT, also referred to as bisulfite Watson / bisulfite Watson reverse) or the original bottom / complement to the original bottom (OB/CTOB, also referred to as bisulfite Crick / bisulfite Crick reverse).

[7]"Redundant" hits are those where at least half of the two alignments overlap.

rightmost position minus the leftmost position[8]. If $|\mathcal{I}| \leq 5000$, the insert size is added to the list of approximate insert sizes. If there are less than ten insert sizes in the list after all reads are processed, then all reads on that thread are considered to be not proper pairs.

If there are ten or more insert sizes, the values are sorted in ascending order and the 25th (p_{25}), 50th (p_{50}), and 75th (p_{75}) percentile values are found. Bounds for ignoring outliers when calculating the mean and standard deviation are then calculated:

$$b_{lo} = \lfloor p_{25} - 2 \times (p_{75} - p_{25}) + 0.499 \rfloor \tag{12}$$

$$b_{hi} = \lfloor p_{75} + 2 \times (p_{75} - p_{25}) + 0.499 \rfloor. \tag{13}$$

Next, the mean, $\mathcal{I}_{avg}$, and standard deviation, $\mathcal{I}_{std}$, for the values falling between $[b_{lo}, b_{hi}]$ are calculated.

Finally, lower, $\mathcal{I}_{min}$, and upper, $\mathcal{I}_{max}$, bounds for proper pair insert sizes are calculated. $\mathcal{I}_{min}$ is defined as the lower of Equations 14 and 15.

$$\mathcal{I}_{min} = \lfloor p_{25} - 3 \times (p_{75} - p_{25}) + 0.499 \rfloor \tag{14}$$

$$\mathcal{I}_{min} = \lfloor \mathcal{I}_{avg} - 4 \times \mathcal{I}_{std} + 0.499 \rfloor \tag{15}$$

$\mathcal{I}_{max}$ is defined as the higher of Equations 16 and 17.

$$\mathcal{I}_{max} = \lfloor p_{75} + 3 \times (p_{75} - p_{25}) + 0.499 \rfloor \tag{16}$$

$$\mathcal{I}_{max} = \lfloor \mathcal{I}_{avg} + 4 \times \mathcal{I}_{std} + 0.499 \rfloor \tag{17}$$

1.3.3 Mapping Quality for Paired-End Alignment

For paired-end alignments with at least one primary alignment in both reads (and no override from the user to skip pairing), a paired mapping quality score is calculated based on proper pairing scores (discussed below) and the the individual Q_{map} scores for each read.

First, pairing scores are calculated for possible proper pairs for the two mates. Briefly, a proper pair is defined as the two mates being on the same bisulfite strand and chromosome and the insert size falls within the bounds of being properly paired, $[\mathcal{I}_{min}, \mathcal{I}_{max}]$ (defined in Section 1.3.2). For those pairs that pass these requirements, a z-score is calculated:

$$z = \frac{(\mathcal{I} - \mathcal{I}_{avg})}{\mathcal{I}_{std}}, \tag{18}$$

where $\mathcal{I}_{avg}$ and $\mathcal{I}_{std}$ are defined in Section 1.3.2. The z-score is then used to calculate the pairing score:

$$s_{pair} = \max\left(0, \left\lfloor \mathcal{S}_1 + \mathcal{S}_2 + \frac{A \times \ln\left[2 \times \text{erfc}\left(|z|/\sqrt{2}\right)\right]}{\ln(4)} + 0.499 \right\rfloor\right), \tag{19}$$

where $\mathcal{S}_1$ and $\mathcal{S}_2$ are the best SW scores for read 1 and read 2, A is defined in Section 1.2 and $\text{erfc}(\cdot)$ is the complementary error function defined as $\text{erfc}(x) = \frac{2}{\sqrt{\pi}} \int_x^\infty e^{-t^2} dt$.

If no pairing scores are calculated, then Q_{map} for each read in the pair is calculated using the procedure laid out in Section 1.3.1. Otherwise, the pairing scores are sorted in ascending

[8] For read 1 on the forward strand and read 2 on the reverse strand, this is (*end position of read* 2) – (*start position of read* 1). For read 1 on the reverse strand and read 2 on the forward strand, this is (*end position of read* 1) – (*start position of read* 2).

order. The two highest scores are taken as the "best" (s_{best}) and "next best" (s_{next}) scores. If there is only one proper pair score calculated, the next best score is set to 0. The number of suboptimal proper pairs is found via:

$$n_{sub} = \sum_{i=0}^{k-2} x_i \tag{20}$$

where k is the number of pairing scores and

$$x_i = \begin{cases} 1 & \text{if } s_{next} - s_i \leq \max(\max(A + B, D_o + D_e), I_o + I_e) \\ 0 & \text{otherwise} \end{cases} \tag{21}$$

where s_i is the pairing score for the i-th pair and A, B, D_o, D_e, I_o, I_e are defined in Section 1.2.

If $s_{best} \leq \mathcal{S}_1 + \mathcal{S}_2 - U$ (U defined in Section 1.2), then the reads are treated as unpaired and Q_{map} is calculated individually for each read. Otherwise, the paired Q_{map} can be calculated for the read pair.

The first step is to calculate q_{pe} based on the proper pair scores:

$$q_{pe} = \left\lfloor \frac{6.02 \times (s_{best} - \max(s_{next}, U))}{A} + 0.499 \right\rfloor, \tag{22}$$

where A and U are defined in Section 1.2. q_{pe} is then adjusted if there are one or more sub-optimal pairing scores:

$$q_{pe} = q_{pe} - \lfloor 4.343 \times \ln(n_{sub} + 1) + 0.499 \rfloor, \tag{23}$$

fixed within the boundaries of 0 and 60:

$$q_{pe} = \begin{cases} 60 & \text{if } q_{pe} > 60 \\ 0 & \text{if } q_{pe} < 0 \\ q_{pe} & \text{otherwise,} \end{cases} \tag{24}$$

and then adjusted by the average fraction of the two alignments covered by repetitive seeds:

$$q_{pe} = \left\lfloor q_{pe} \times \left(1.0 - \frac{\phi_1 + \phi_2}{2}\right) + 0.499 \right\rfloor, \tag{25}$$

where ϕ_i is the fraction of alignment $i \in \{1, 2\}$ covered by repetitive seeds.

Individual Q_{map} scores are then calculated for read 1 (Q_1) and read 2 (Q_2) following the procedure in Section 1.3.1. These scores are then adjusted and capped in the following way:

1. Adjust the individual mapping quality scores by adding 40 to the calculated score with a cap at q_{pe}:

$$Q_i = \max[Q_i,\ \min(q_{pe},\ Q_i + 40)] \tag{26}$$

2. Cap the mapping quality score at the tandem repeat score:

$$Q_i = \min(Q_i,\ \lfloor 6.02 \times (\mathcal{S} - s_{tandem})/A + 0.499 \rfloor) \tag{27}$$

where $i \in \{1, 2\}$. The Q_i calculated in Equation 27 are the paired mapping quality scores for reads 1 and 2 used in BISCUIT.

2 Supplementary Methods

Methods used in producing data are described below. For examples of the code used, see https://github.com/huishenlab/biscuit_paper_code.

2.1 Alignment Validation

Data for the alignment validation are from ten TruSeq Methyl Capture EPIC datasets available on SRA (Supplemental Table S1) [2]. Read trimming was applied using TrimGalore! (https://github.com/FelixKrueger/TrimGalore, version 0.6.6 with cutadapt version 3.2):

```
trim_galore \
    --no_report_file \
    --basename sample_XXXM \
    --cores 4 \
    --paired read1.fq.gz read2.fq.gz
```

The manufacturer's manifest (https://support.illumina.com/downloads/truseq-methyl-capture-epic-manifest-file.html) for the on-target region set was downloaded and lifted over from hg19 to hg38 using the UCSC site: https://genome.ucsc.edu/cgi-bin/hgLiftOver.

Each dataset was aligned to hg38 with no contigs. Genome indexes were created for each aligner in the following manner:

```
# BISCUIT
biscuit index hg38.fa

# Bismark
bismark_genome_preparation genome_dir

# BSBolt
bsbolt Index -G hg38.fa -DB hg38

# bwa-meth
bwameth.py index hg38.fa

# gemBS
gemBS prepare --config hg38.conf --text-metadata hg38.samp.conf
gemBS index
```

The BISCUIT, Bismark, BSBolt, bwa-meth and gemBS pipelines, as detailed below, follow best practices for analysis with each toolkit. Alignments performed with BISCUIT used the following pipeline:

```
# align, mark dups, sort
( time \
    biscuit align -@ 30 -b 1 hg38.fa ${FQ1} ${FQ2} | \
    dupsifter hg38.fa | \
    samtools sort -@ 30 -m 5G -o ${BAM} -O BAM -
)
```

```
# index
( time \
    samtools index -@ 30 ${BAM}
)

# pileup
( time \
    biscuit pileup -@ 30 -o ${VCF} hg38.fa ${BAM}
)

( time \
    bgzip -@ 30 ${VCF}
)

( time \
    tabix -p vcf ${VCF}.gz
)

# vcf2bed
( time \
    biscuit vcf2bed ${VCF}.gz | gzip > ${BED}.gz
)
```

where BISCUIT version 1.2.1, samtools version 1.17, and dupsifter version 1.1.1 were used. FQ1, FQ2, BAM, VCF, and BED represent the sample-specific names used for each aligned sample.

Alignments performed with Bismark (with Bowtie2 version 2.5.1) used the following pipeline:

```
# align
( time \
    bismark \
        --parallel 10 \
        --output_dir ${OUT} \
        --temp_dir ${OUT} \
        --samtools_path ${SAMTOOLS} \
        --genome hg38 \
        -1 ${FQ1} \
        -2 ${FQ2}
)

# dedup
( time \
    deduplicate_bismark \
        --output_dir ${OUT} \
        ${BAM}
)
```

```
# methylation extraction
( time \
    bismark_methylation_extractor \
        --multicore 10 \
        --bedGraph \
        --gzip \
        --output ${OUT} \
        ${DUP}
)
```

where Bismark version 0.24.0 and samtools version 1.17 (SAMTOOLS is the path to the samtools installation) were used. FQ1, FQ2, OUT, BAM, and DUP are sample-specific output file names.

Alignments performed with BSBolt used the following pipeline:

```
# align
( time \
    bsbolt Align -t 30 -DB hg38 -F1 ${FQ1} -F2 ${FQ2} -O ${UNS}
)

# fixmates to prepare for duplicate removal
# use -p to disable proper pair check
( time \
    samtools fixmate -p -m ${UNS}.bam ${FIX}.bam
)

# sort bam by coordinates for duplicate calling
( time \
    samtools sort -@ 30 -o ${SRT}.bam ${FIX}.bam
)

# remove duplicate reads
( time \
    samtools markdup ${SRT}.bam ${BAM}
)

# index bam file for methylation calling
( time \
    samtools index -@ 30 ${BAM}
)

# methylation calling
( time \
    bsbolt CallMethylation \
        -I ${BAM} \
        -DB hg38 \
        -O ${BAS} \
        -BG -CG -t 30
```

```
)
```

where BSBolt version 1.6.0 and samtools version 1.17 were used. FQ1, FQ2, UNS, FIX, SRT, BAM, and BAS are sample-specific output file names.

Alignments performed with bwa-meth used the following pipeline:

```
# align
( time \
    bwameth.py --threads 30 \
        --reference hg38.fa \
        ${FQ1} \
        ${FQ2} | \
    samtools view -o ${UNS} -O BAM -
)

# sort
( time \
    samtools sort -@ 30 -m 5G -o ${SRT} -O BAM ${UNS}
)

# index
( time \
    samtools index -@ 30 ${SRT}
)

# mark duplicates
( time \
    java -Xms8g -Xmx100g -Djava.io.tmpdir=${OUT} -jar \
        $PICARD MarkDuplicates \
        I=${SRT} \
        O=${BAM} \
        M=${PIC} \
        REMOVE_DUPLICATES=false \
        ASSUME_SORTED=true
)

# index (again)
( time \
    samtools index -@ 30 ${BAM}
)

# methlation extraction
( time \
    MethylDackel extract -@ 30 \
        hg38.fa \
        ${BAM}
)
```

where bwa-meth version 0.2.6, samtools version 1.17, picard version 2.27.5, and MethylDackel version 0.6.1 were used. FQ1, FQ2, UNS, SRT, OUT, BAM, and PIC are sample-specific output file names.

Alignments performed with gemBS used the following pipeline:

```
# prepare gemBS inputs
( time \
    gemBS prepare -c ${CON} -t ${SAP}
)

# align
( time \
    gemBS map --tmp-dir ${OUT}
)

# pileup
( time \
    gemBS call --tmp-dir ${OUT}
)

# methylation extraction
( time \
    gemBS extract
)
```

where gemBS version 4.0.4 was used. OUT is a sample-specific file name. CON and SAP are the configuration and sample metadata files. For examples of these files see `https://github.com/huishenlab/biscuit_paper_code/tree/main/speed_bench_bulk`.

The number of reads in the read 1 FASTQ file for each sample was used as the sample's total number of reads. The number of mapped and optimally mapped reads for each aligner was found using these commands (due to Bismark changing the read name when aligning, it had a slightly different command used, as shown):

```
# Mapped reads (BISCUIT, BSBolt, bwa-meth, gemBS)
samtools view -F 2308 ${BAM} | \
cut -f 1 | \
sort -k1 -T ${OUT} | \
uniq | \
wc -l

# Optimally mapped reads (BISCUIT, BSBolt, bwa-meth, gemBS)
samtools view -F 2308 -q 40 ${BAM} | \
cut -f 1 | \
sort -k1 -T ${OUT} | \
uniq | \
wc -l

# Mapped reads (Bismark)
samtools view -F 2308 ${BAM} | \
```

```
cut -f 1 | \
awk -F"_" '{ print $1 }' | \
sort -k1 -T ${OUT} | \
uniq | \
wc -l

# Optimally mapped reads (Bismark)
samtools view -F 2308 -q 40 ${BAM} | \
cut -f 1 | \
awk -F"_" '{ print $1 }' | \
sort -k1 -T ${OUT} | \
uniq | \
wc -l
```

where samtools version 1.17 was used and BAM and OUT are sample-specific output file names.

Reads that were on-target were determined by intersecting the aligned BAM with the manufacturer's manifest file using bedtools (version 2.30.0) and samtools (version 1.17):

```
# Find on target optimally mapped reads
samtools view -hbu -F 2308 -q 40 ${BAM} | \
bedtools intersect -wa -a stdin -b ${FST} | \
samtools view | \
cut -f 1 | \
sort -k1 -T ${OUT} | \
uniq | \
gzip \
> ${ONN}
```

where BAM, OUT, and ONN are sample-specific output file names and FST is the manifest file. Bismark required the `awk` command used in finding the number of mapped reads (not shown for brevity).

2.2 Speed Benchmarking

Data for the speed benchmarking are available on SRA and come from human, mouse, and zebrafish samples across different tissues and disease states using both traditional WGBS and the more recent EM-seq (Supplemental Table S2) [3, 6, 7, 10–12]. For each dataset, the FASTQ files were subsampled to 1, 5, 10, 25, 50, 100, and 250 million reads using seqtk (`https://github.com/lh3/seqtk`, version 1.3-r113-dirty):

```
seqtk sample reads.fq.gz NNN | \
gzip > reads_NNN.fq.gz
```

where NNN is the number of reads subsampled. It should be noted for the zebrafish datasets that multiple samples were combined for individual datasets in order to reach a sizeable number of reads to subsample. Further, one zebrafish dataset, even after combining, only had enough reads to subsample to 1, 5, 25, and 50 million reads. Apart from the two TCGA samples, which were already trimmed, read trimming was applied using the same process as in Section 2.1.

Human datasets were aligned to hg38 with no contigs, while mouse datasets were aligned to mm10 with no contigs. Zebrafish datasets were aligned to z11 with contigs. Genome indexes created for alignment validation were used for speed benchmarking. The time to create the genome indexes was not included in the benchmarking times presented. For each time point collected, GNU time (version 1.9) was used.

For speed benchmarking, the same pipelines described for the alignment validation were used. In general, the alignment time is the amount of time needed to get a BAM that is duplicate marked, sorted, and indexed, while the methylation extraction time is the time to extract methylation from the sorted BAM. The end-to-end time is the sum of the alignment and methylation extraction times.

The BISCUIT alignment time is sum of the time to run the biscuitSifter pipeline (BISCUIT, dupsifter, and samtools) and the indexing time. The methylation extraction time is the sum of the pileup, bgzip, tabix, and vcf2bed times.

The Bismark alignment time is the sum of the align and deduplication times, while the methylation extraction time is the time to run `bismark_methylation_extractor`. Bismark does not need to be sorted in order to extract methylation, so sorting and indexing was not included.

The BSBolt alignment time is the time to align with BSBolt and fix mates, sort, mark duplicates, and index with samtools. The methylation extraction time is the time to call methylation with BSBolt.

The bwa-meth alignment time is the sum of the alignment, sort, duplicate marking, and two indexing times. One index is needed for marking duplicates with Picard, while the second is needed to index the duplicate marked BAM output from Picard. The methylation extraction time is the time to extract methylation with MethylDackel.

The gemBS alignment time is the time to prepare and map with gemBS. The methylation extraction time is the time to call and extract methylation.

2.3 Single-cell WGBS Alignments

Two different single-cell WGBS datasets were used for this analysis. 249 single cells (153 human cells and 96 mouse cells) were taken from snmc-seq2 (GEO accession number GSE112471) [4] and 49 mouse cells from the Smallwood et al. protocol paper (GEO accession number GSE56879, only oocytes and embryonic stem cells were used) [8] were downloaded from SRA. The Smallwood et al. data was not trimmed, while the snmc-seq2 data was trimmed to remove barcodes and Adaptase bases from reads 1 and 2 with cutadapt and compressed with pigz:

```
cutadapt --report=minimal -Z \
    -u 6 -U 14 \
    -o SAMP_trimmed_R1.fastq \
    -p SAMP_trimmed_R2.fastq \
    SAMP_1.fastq.gz SAMP_2.fastq.gz \
> SAMP_report.txt 2>&1

pigz -p 8 SAMP_trimmed_R1.fastq
pigz -p 8 SAMP_trimmed_R2.fastq
```

where `SAMP` represents the SRA accession ID for each cell in the dataset.

The BISCUIT pipeline for both snmc-seq2 and Smallwood et al. followed the same pipeline used in the alignment validation with two small alterations: dropping `-b 1` from the `biscuit align` invocation and adding `-k 1` to the `biscuit vcf2bed` call. For both protocols, the BSBolt and gemBS pipelines also followed the corresponding alignment validation pipelines with each aligner adding the respective option to allow for non-directional alignment (`-UN` in `BSBolt Align` and `--read-non-stranded` in `gemBS map`). The bwa-meth snmc-seq2 and Smallwood et al. pipelines followed the alignment validation pipeline for bwa-meth with no changes.

Rather than following the same pipeline as the alignment validation, Bismark followed the respective pipelines used in the publication of each single-cell WGBS protocol. Each protocol uses a slightly different alignment call, but then followed a similar pipeline thereafter. In Smallwood et al., read 1 and read 2 were aligned with:

```
# Read 1
( time \
    bismark \
    --non_directional \
    --parallel 10 \
    --output_dir ${OUT} \
    --temp_dir ${OUT} \
    --samtools_path ${SAMTOOLS} \
    --genome mm10 \
    ${FQ1}

# Read 2
( time \
    bismark \
    --non_directional \
    --parallel 10 \
    --output_dir ${OUT} \
    --temp_dir ${OUT} \
    --samtools_path ${SAMTOOLS} \
    --genome mm10 \
    ${FQ2}
```

In snmc-seq2, read 1 and read 2 were aligned with:

```
# Read 1
( time \
    bismark \
    --pbat \
    --parallel 10 \
    --output_dir ${OUT} \
    --temp_dir ${OUT} \
    --samtools_path ${SAMTOOLS} \
    --genome ${REF} \
    ${FQ1}
)
```

```
# Read 2
( time \
    bismark \
    --parallel 10 \
    --output_dir ${OUT} \
    --temp_dir ${OUT} \
    --samtools_path ${SAMTOOLS} \
    --genome ${REF} \
    ${FQ2}
)
```

For both protocols, SAMTOOLS is the path to the samtools installation and OUT, FQ1, FQ2, and REF are sample-specific file names.

After alignment, both protocols used the following pipline:

```
# deduplication
( time \
    deduplication_bismark \
    --output_dir ${OUT} \
    ${BAM1}
)

( time \
    deduplication_bismark \
    --output_dir ${OUT} \
    ${BAM2}
)

# name sort for merging
( time \
    samtools sort -n -@ 30 \
    -o ${SRT1} -O BAM \
    ${DUP1}
)

( time \
    samtools sort -n -@ 30 \
    -o ${SRT2} -O BAM \
    ${DUP2}
)

# merge
( time \
    samtools merge -n -@ 30 -O BAM \
    ${MERGE} \
    ${SRT1} \
    ${SRT2}
)
```

```
# methylation extraction
( time
    bismark_methylation_extractor \
    --multicore 10 \
    --bedGraph \
    --gzip \
    --output ${OUT} \
    ${MERGE}
)
```

Here, OUT, BAM1, BAM2, SRT1, SRT2, and MERGE are sample-specific output file names.

Read counts for BISCUIT, BSBolt, bwa-meth, and gemBS were found in the same manner as the alignment validation. The Bismark results extracted the read names from the individual read BAMS, found the unique read names across both, then performed counting in the same manner as the alignment validation.

2.4 Structural Variant Discovery

Sequencing data were downloaded from SRA (accession number SRR1800202) [9]. The FASTQ files were processed with BISCUIT version 1.1.0 and dupsifter version 1.0.0:

```
biscuit align \
    -@ 30 -b 1 -M -R 'read group' \
    hg38.fa \
    read1.fq.gz \
    read2.fq.gz | \
dupsifter hg38.fa | \
samtools sort -@ 8 -m 5G -o output.bam -O BAM -

samtools index -@ 15 output.bam

biscuit pileup \
    -@ 30 hg38.fa \
    output.bam | \
bgzip > pileup.vcf.gz
tabix -p vcf pileup.vcf.gz

biscuit vcf2bed pileup.vcf.gz | \
biscuit mergecg hg38.fa - | \
bgzip > mergecg.bed.gz
tabix -p bed mergecg.bed.gz
```

Structural variants were called using manta version 1.6.0 and lumpy version 0.2.13. Manta was run in tumor-only analysis mode using:

```
(manta install path)/bin/configManta.py \
    --tumorBam output.bam \
    --referenceFasta hg38.fa \
```

```
    --runDir (manta output directory) \
    --callRegions regions.bed.gz
```

The call regions were determined by taking the inverse of the ENCODE hg38 exclusion list BED file, restricting to the primary chromosomes, and removing the mitochondrial chromosome. The workflow was then run with `python runWorkflow.py` in the output directory created by running the configuration script. Structural variants with lumpy were found via:

```
lumpyexpress \
    -B output.bam \
    -o output.lumpy.vcf

gzip output.lumpy.vcf
```

2.5 SNV Validation and Precision-Recall Curves

We used WGS data from Genome-in-a-Bottle (GIAB) and WGBS data [1] performed on the GM12878 cell line to validate single nucleotide variant calling with BISCUIT. WGBS FASTQ files for two replicates were downloaded from SRA (SRA accession numbers SRR4235788 and SRR4235789) and trimmed using TrimGalore! (version 0.6.6 with cutadapt 4.1) and subsampled to 500 million reads each. The subsampled FASTQs were then aligned to hg38 using:

```
biscuit align \
    -@ 30 -b 1 -R ‘(read group)’ \
    hg38.fa \
    read1.fq.gz \
    read2.fq.gz | \
dupsifter --add-mate-tags hg38.fa | \
samtools sort -@ 8 -m 5G -o output.bam -O BAM -
```

The BAM was then indexed and a pileup VCF created:

```
samtools index output.bam

biscuit pileup -@ 30 \
    hg38.fa \
    output.bam \
    -o pileup.vcf
bgzip -@ 20 pileup.vcf
tabix -p vcf pileup.vcf.gz
```

After the VCFs were created for each replicate, the intersection between the two was found using:

```
bcftools isec rep1.pileup.vcf.gz rep2.pileup.vcf.gz
```

SNVs in replicate 1 (SRR4235788) that were found in both replicates were then filtered to remove SNVs with low genotype quality (GQ ≤ 5), that were not on the canonical chromosomes, or had a genotype of 0/0 relative to the reference. The resulting set of variants were used as the BISCUIT (i.e., WGBS) variants in the validation.

Two different datasets were used for GIAB. First, insertions and deletions were filtered from the high confidence variants VCF for NA12878 using vcftools version 0.1.16, leaving only the high confidence SNVs. These SNVs were used as the full GIAB set of variants during the validation process. Second, GIAB combines many types of sequencing technologies. Therefore, the Illumina-only FASTQ files were downloaded (the full list is available at `github.com/genome-in-a-bottle/giab_data_indexes/blob/master/NA12878/sequence.index.NA12878_Illumina300X_wgs_09252015`) to better compare with the Illumina-generated WGBS replicates. The individual FASTQ files were combined into a single file for reads 1 and 2 using pigz version 2.4 and then downsampled to 500 million reads using seqtk. The downsampled FASTQ files were then aligned with BWA-MEM (version 0.7.17-r1188) via:

```
bwa mem -t 30 -R '(read group)' \
    hg38.fa \
    read1.fq.gz \
    read2.fq.gz | \
samblaster --addMateTags | \
samtools sort -@ 8 m 5G -o output.bam -O BAM -
```

Samblaster (version 0.1.26) was used to mark duplicates. Variants were found using GATK (version 4.1.4.1). Within GATK, the aligned BAMs had base quality recalibration applied using BaseRecalibrator and ApplyBQSR, then germline variants were found using HaplotypeCaller. SNVs were extracted using SelectVariants and then filtered based on GATK best practices using VariantFiltration. Additionally, variants not on canonical chromosomes or with a genotype of 0/0 relative to the reference were filtered to better match with BISCUIT. Note, GATK best practices has a stricter variant quality cutoff, so no additional filtering was applied for genotype quality. The variants that passed both sets of filters were used as the GATK (i.e., WGS) variants during validation.

Once the three sets of variants had been found, the three-way intersection between the sets was found using:

```
bcftools isec -n+1 \
    biscuit.vcf.gz \
    gatk.vcf.gz \
    giab.vcf.gz
```

To create the precision-recall curves, the BISCUIT and GIAB SNV data described above were restricted to the first 22 megabases of chromosome 11, phased haplotypes were converted to unphased, then intersected with the inverse of the ENCODE exclusion list and dbSNP (version 153) common SNPs. Precision and recall were calculated using the GIAB dataset as the ground truth. The curve labeled as "GQ >= n" is drawn from the results as is, with no additional filtering applied. The curve labelled "GQ >= 15 (dbSNP+) / GQ >= n (dbSNP-)" has an added filter where SNVs that intersect common dbSNP SNPs with a minor allele frequency (MAF) greater than 0.05 were allowed a GQ greater than or equal to 15, whereas all other variants were greater than or equal to n.

Based on the precision-recall curves, it was determined that a filter using a dbSNP prior would improve the false positive rate of BISCUIT SNV calling. To create the filtered BISCUIT SNVs, an additional filter was added to the previously described BISCUIT SNV calls. The filter was that SNVs had to fall in the inverse of the ENCODE exclusion list and either intersect a common dbSNP SNP with a MAF ≥ 0.05 and genotype quality ≥ 15 or, if not,

it must have a genotype quality ≥ 60. This newly filtered set of variants was then used for the final, filtered set of BISCUIT SNVs.

2.6 EpiBED and Allele-Specific Methylation

FASTQs from Morrison et al. [5] were used, specifically the two sample A technical replicates for the high-input New England Biolabs kit and the two sample A technical replicates for the high-input Swift Biosciences kit. The data was aligned as described in [5], then each kit's technical replicates were merged using:

```
samtools merge -@ 20 \
    -o A(kit).bam \
    (kit)1.bam \
    (kit)2.bam
samtools index -@ 20 A(kit).bam
```

where (kit) is either "neb" or "swift." Each kit's data were then passed through the following pipeline to create individual epiBED files:

```
biscuit pileup \
    -g chr15:24954913-24955009 \
    -@ 40 \
    hg38.fa \
    A(kit).bam | \
biscuit vcf2bed -t snp -k 1 - \
> A(kit).snp.bed

biscuit epiread \
    -g chr15:24954913-24955009 \
    -@ 40 \
    -B A(kit).snp.bed \
    hg38.fa \
    A(kit).bam | \
sort -k1,1 -k2,2n > A(kit).epibed

bgzip A(kit).epibed
```

The resulting epiBED files were merged into a single epiBED file for generating the figure

```
zcat Aneb.epibed.gz Aswift.epibed.gz | \
sort -k1,1 -k2,2n | \
uniq | \
bgzip > Asample.epibed.gz

tabix -p bed Asample.epibed.gz
```

The region was selected based on annotations for imprinted CpG probes in the EPIC methylation array, specifically the SNRPN-SNURF imprinted region. After creating the epiBED file, it was imported into R using biscuiteer (version 1.13.1) with the readEpibed function. The figure was created using bisplotti (version 0.0.19, https://github.com/huishenlab/

bisplotti) with tabulateEpibed and plotEpibed. Reads were subsetted to those that covered both the SNP and CpG and sorted based on their methylation status at the EPIC probe CpG location.

3 Supplementary Tables

ID	Species	Tissue	Cell Line	Sequencing Technology	Number of Reads (millions)	Citation
SRR13051114	Human	B-lymphocyte	NA24695 / HG007	TruSeq Methyl Capture EPIC	60.7	[2]
SRR13051115	Human	B-lymphocyte	NA24695 / HG007	TruSeq Methyl Capture EPIC	58.6	[2]
SRR13051116	Human	B-lymphocyte	NA24694 / HG006	TruSeq Methyl Capture EPIC	66.4	[2]
SRR13051117	Human	B-lymphocyte	NA24694 / HG006	TruSeq Methyl Capture EPIC	61.8	[2]
SRR13051118	Human	B-lymphocyte	NA24631 / HG005	TruSeq Methyl Capture EPIC	80.4	[2]
SRR13051120	Human	B-lymphocyte	NA24631 / HG005	TruSeq Methyl Capture EPIC	60.9	[2]
SRR13051121	Human	B-lymphocyte	NA24143 / HG004	TruSeq Methyl Capture EPIC	84.7	[2]
SRR13051122	Human	B-lymphocyte	NA24143 / HG004	TruSeq Methyl Capture EPIC	58.7	[2]
SRR13051123	Human	B-lymphocyte	NA24149 / HG003	TruSeq Methyl Capture EPIC	84.3	[2]
SRR13051124	Human	B-lymphocyte	NA12878 / HG001	TruSeq Methyl Capture EPIC	81.6	[2]

Supplemental Table S1: **Datasets used for the alignment validation of BISCUIT against other cytosine-conversion-aware aligners.** All datasets are available on SRA and are part of the Genome-in-a-Bottle dataset.

ID	Species	Tissue	Cell Line	Sequencing Technology	Number of Reads (Millions)	Citation	Notes
SRR13076816	Human	cfDNA	–	EM-seq	446.8	[10]	–
SRR13076817	Human	cfDNA	–	Em-seq	409.8	[10]	–
SRR4235788	Human	Lymphoblastoid	GM12878	WGBS	619.3	[1]	Used in variant validation
SRR4235789	Human	Lymphoblastoid	GM12878	WGBS	569.5	[1]	Used in variant validation
TCGA_LUSC_2600	Human	Bronchus and lung	–	WGBS	563	[12]	Lung squamous cell carcinoma
TCGA_COAD_A00R	Human	Colon	–	WGBS	637	[12]	Colon adenocarcinoma
ERR5005148	Zebrafish	Brain	–	WGBS	120.4	[6]	Combined into ERR5005148_ERR5005151
ERR5005149	Zebrafish	Brain	–	WGBS	93.6	[6]	Combined into ERR5005148_ERR5005151
ERR5005150	Zebrafish	Brain	–	WGBS	88.4	[6]	Combined into ERR5005148_ERR5005151
ERR5005151	Zebrafish	Brain	–	WGBS	106.9	[6]	Combined into ERR5005148_ERR5005151
SRR11614917	Zebrafish	Whole embryo	–	EM-seq	17.4	[7]	Combined into SRR11614917_SRR11614920
SRR11614918	Zebrafish	Whole embryo	–	EM-seq	19.3	[7]	Combined into SRR11614917_SRR11614920
SRR11614919	Zebrafish	Whole embryo	–	EM-seq	21	[7]	Combined into SRR11614917_SRR11614920
SRR11614920	Zebrafish	Whole embryo	–	EM-seq	23.1	[7]	Combined into SRR11614917_SRR11614920
SRR12797058	Zebrafish	Heart	–	WGBS	50.4	[7]	Combined into SRR12797058_SRR12797065
SRR12797059	Zebrafish	Heart	–	WGBS	57.1	[7]	Combined into SRR12797058_SRR12797065
SRR12797060	Zebrafish	Intestine	–	WGBS	54	[7]	Combined into SRR12797058_SRR12797065
SRR12797061	Zebrafish	Intestine	–	WGBS	54.4	[7]	Combined into SRR12797058_SRR12797065
SRR12797062	Zebrafish	Skin	–	WGBS	59.2	[7]	Combined into SRR12797058_SRR12797065
SRR12797063	Zebrafish	Skin	–	WGBS	41.4	[7]	Combined into SRR12797058_SRR12797065
SRR12797064	Zebrafish	Muscle	–	WGBS	67	[7]	Combined into SRR12797058_SRR12797065
SRR12797065	Zebrafish	Muscle	–	WGBS	50.9	[7]	Combined into SRR12797058_SRR12797065
SRR13482506	Mouse	Total embryo	–	WGBS	442.8	[11]	–
SRR13482508	Mouse	Total embryo	–	WGBS	440.3	[11]	–

Supplemental Table S2: **Datasets used for the speed benchmarking of BISCUIT against other cytosine-conversion-aware aligners.** All datasets are available on SRA, with the exception of the TCGA samples, which are available on the NCI GDC legacy Data Portal. Even after merging, SRA accession IDs SRR11614917, SRR11614918, SRR11614919, and SRR11614920 only had enough reads to subsample up to 50 million reads. All other samples (or merged samples) could subsample up to 250 million reads.

Sample	Alignment attempted?	Completed?	Sample	Alignment attempted?	Completed?
SRR1248444	yes	–	SRR1248469	yes	–
SRR1248445	yes	–	SRR1248477	yes	–
SRR1248446	yes	–	SRR1248478	yes	–
SRR1248447	yes	yes	SRR1248479	yes	–
SRR1248448	yes	–	SRR1248480	yes	yes
SRR1248449	yes	–	SRR1248481	–	–
SRR1248450	yes	yes	SRR1248482	–	–
SRR1248451	yes	–	SRR1248483	–	–
SRR1248452	yes	yes	SRR1248484	–	–
SRR1248453	yes	–	SRR1248485	–	–
SRR1248454	yes	–	SRR1248486	–	–
SRR1248455	yes	yes	SRR1248487	–	–
SRR1248456	yes	yes	SRR1248488	–	–
SRR1248457	yes	yes	SRR1248489	–	–
SRR1248458	yes	–	SRR1248490	–	–
SRR1248459	yes	yes	SRR1248491	–	–
SRR1248460	yes	–	SRR1248492	–	–
SRR1248461	yes	–	SRR1248493	–	–
SRR1248462	yes	–	SRR1248494	–	–
SRR1248463	yes	yes	SRR1248495	–	–
SRR1248464	yes	yes	SRR1248496	–	–
SRR1248465	yes	yes	SRR1248497	–	–
SRR1248466	yes	yes	SRR1411188	–	–
SRR1248467	yes	–	SRR1411189	–	–
SRR1248468	yes	–			

Supplemental Table S3: **SRA accession IDs for the Smallwood et al. single-cell WGBS protocol.** All samples completed alignments for BISCUIT, Bismark, BSBolt, and bwa-meth. Samples where alignments were attempted with gemBS are marked as "yes" under "Alignment attempted?". Those that completed in less than two days are marked as "yes" under "Completed?". Samples that either weren't attempted or did not finish in two days are denoted by "–".

Column	1	2	3	4	5	6	7	8	9
Description	Chromosome	Start	End	Read Name	Read Number in Pair	BS Strand	CpG RLE String	GpC RLE String	Variant RLE String
WGBS Example	chr1	0	102	read1	1	+	F3x59Ux18UxUx15F3	.	F3x94GF3
NOMe-seq Example	chr2	100	200	read2	1	+	F3x62Mx21Mx9F3	F3x30Sx49Sx13F3	F3x41Ax52F3

Supplemental Table S4: **The epiBED format simultaneously captures genetic and epigenetic information in a compact, standards-compliant format.** The columns are: 1) Chromosome, 2) Zero-based start location of read, 3) One-based non-inclusive end location of read, 4) Name of read, 5) Read number in paired-end sequencing, 6) Bisulfite strand ("+" for OT/CTOT strand / "-" for OB/CTOB strand), 7) Run-length encoded string for CpG methylation, 8) Run-length encoded string for GpC methylation, and 9) Run-length encoded string for SNPs and indels. Columns 1 — 3 come directly from the BED specification. Columns 8 – 9 will contain a "." if not found. Also provided are example epiBED entries for both WGBS and NOMe-seq.

4 Supplementary Figures

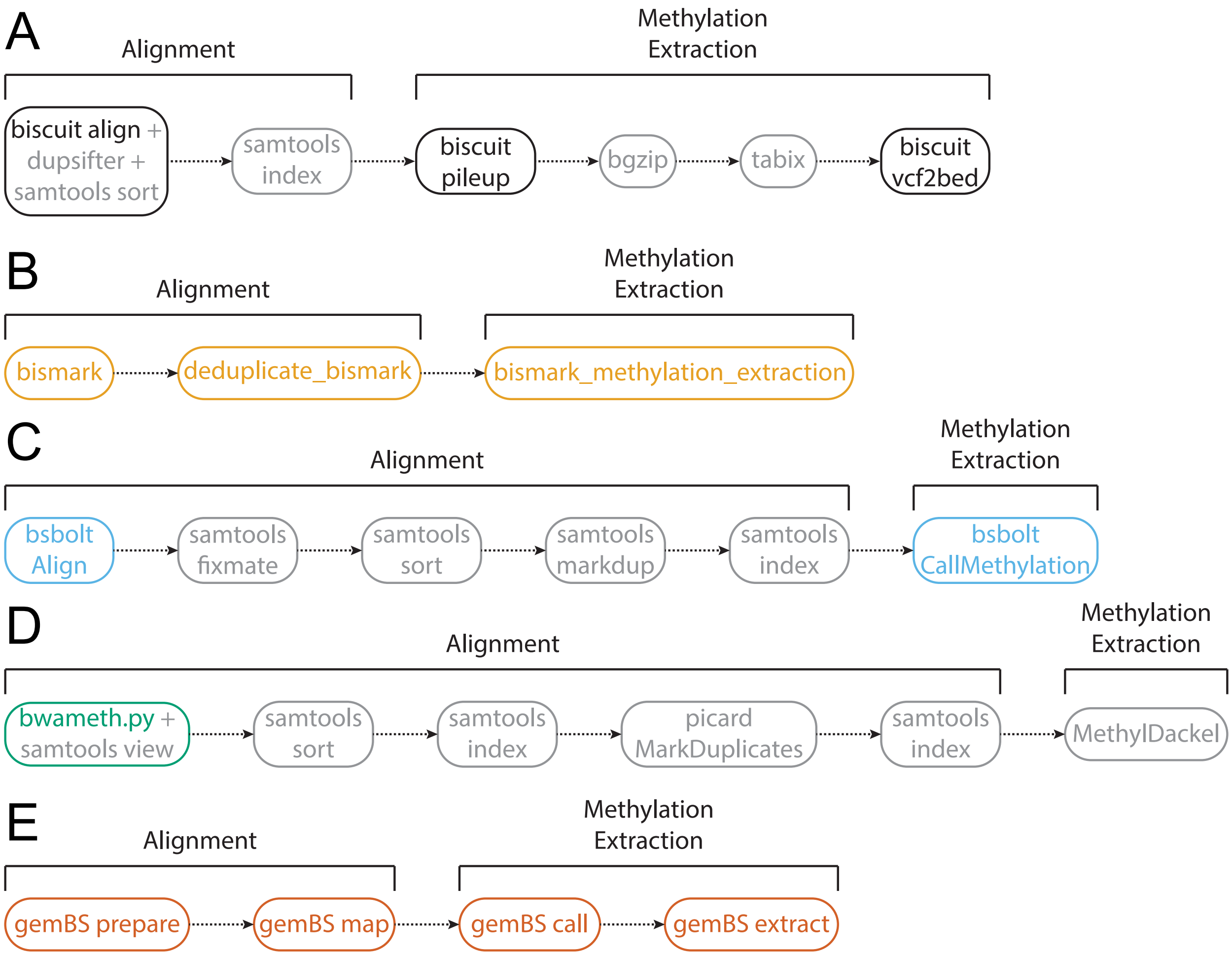

Supplemental Figure S1: **Analysis pipelines follow best practices for benchmarked aligners as suggested by tool maintainers.** Pipelines are shown for (A) BISCUIT, (B) Bismark, (C) BSBolt, (D) bwa-meth, and (E) gemBS. Steps considered under the "Alignment Time" are marked by "Alignment." Steps for the "Methylation Extraction Time" are marked by "Methylation Extraction." Colored text denotes tools or subcommands provided by the given aligner, while light gray text denotes third-party tools used to perform specific tasks (e.g., duplicate marking or coordinate sorting).

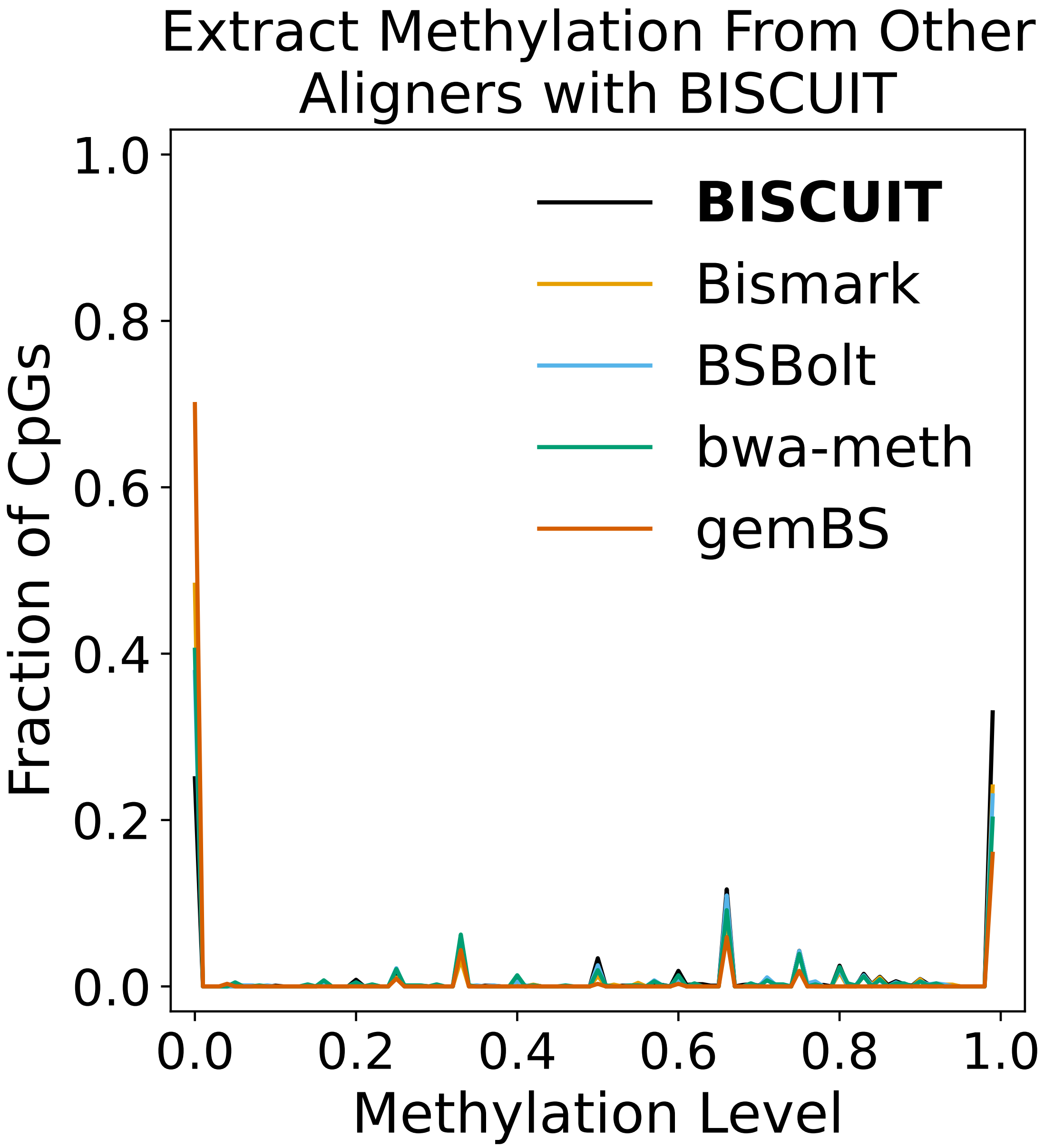

Supplemental Figure S2: **BISCUIT is able to process BAMs aligned by other aligners.** Data is mapped and duplicate marked following each aligner's suggested pipeline, with duplicates being marked with Picard for gemBS as duplicate marking is built into BScall. The aligned and duplicate marked BAMs are then passed through BISCUIT to extract methylation.

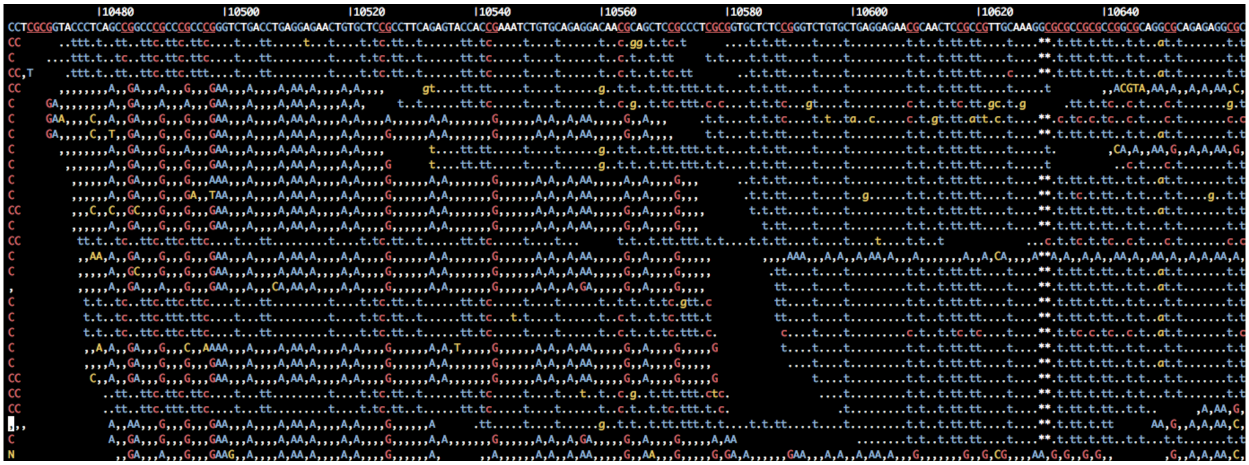

Supplemental Figure S3: **BISCUIT provides functionality to view methylation and mutation status simultaneously.** CpGs in the reference (top line of bases) are marked in red, while other C's and G's are marked in blue. For reads (all lines below the reference), methylated C's (or G's from the OB/CTOB strand) are marked in red, whereas unmethylated C's (or G's) are marked in blue. Mismatches that are not related to cytosine conversion are marked in yellow.

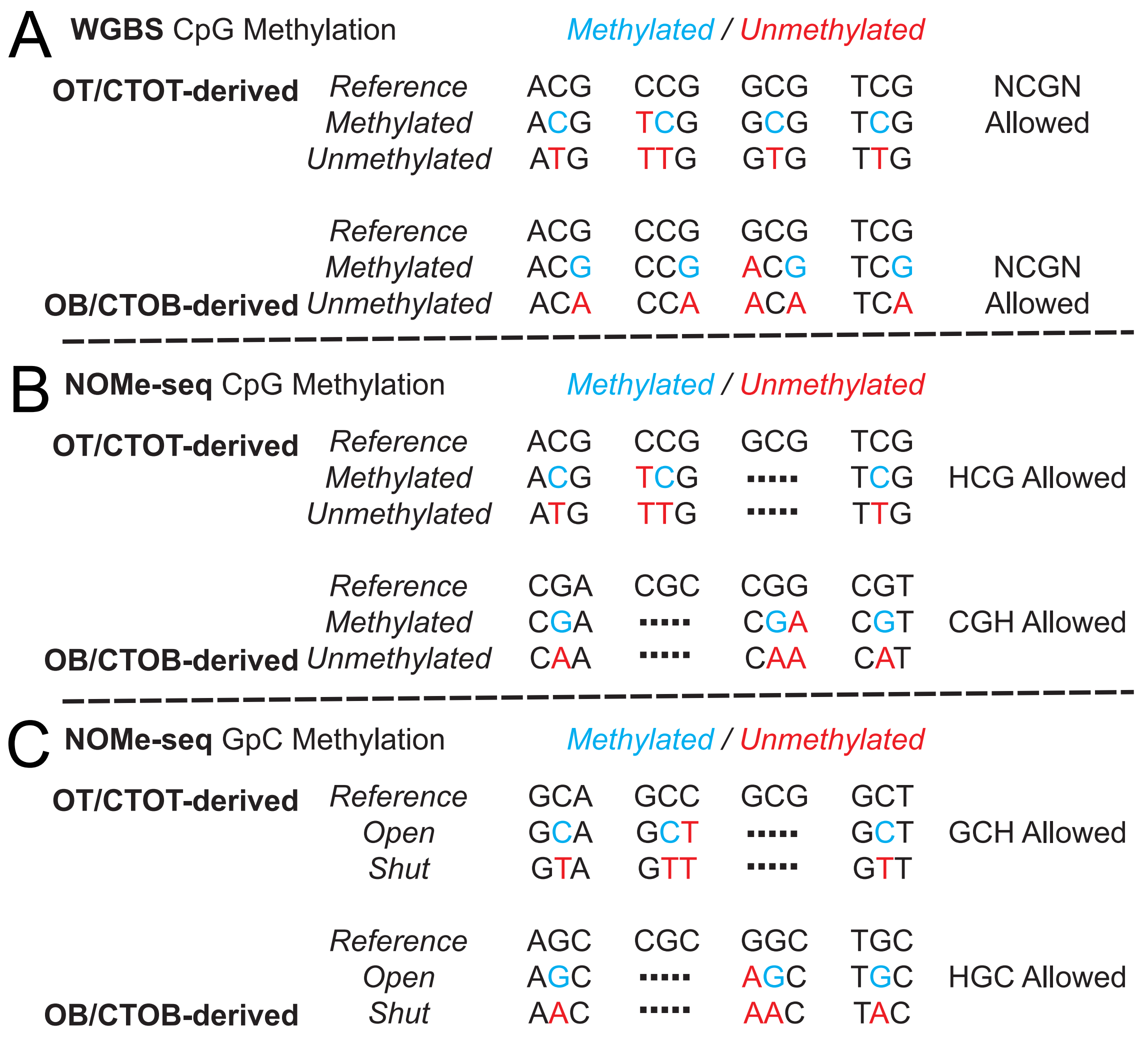

Supplemental Figure S4: **WGBS and NOMe-seq have different three-base contexts that are allowed when determining methylation and accessibility.** (A) CpG methylation in WGBS is allowed in any CpG context. Reads deriving from the OT/CTOT strand determine the methylation state from the C (C = methylated, T = unmethylated), while reads deriving from the OB/CTOB strand use the G (G = methylated, A = unmethylated). (B) CpG methylation in NOMe-seq mode allows CpGs only in the HCG (OT/CTOT) or CGH (OB/CTOB) context. This avoids ambiguity in the GCG (or CGC) context as the C (or G) can be methylated either naturally or due to the GpC methyltransferase used in NOMe-seq. The methylation state is determined in the same manner as WGBS, but with the added context restriction. (C) GpC accessibility in NOMe-seq mode allows GpCs only in the GCH (OT/CTOT) or HGC (OB/CTOB) context to avoid ambiguous methylation. Accessibility is determined using the C (C = open, T = shut) for reads deriving from the OT/CTOT strand or the G (G = open, A = shut) for reads deriving from the OT/CTOB strand.

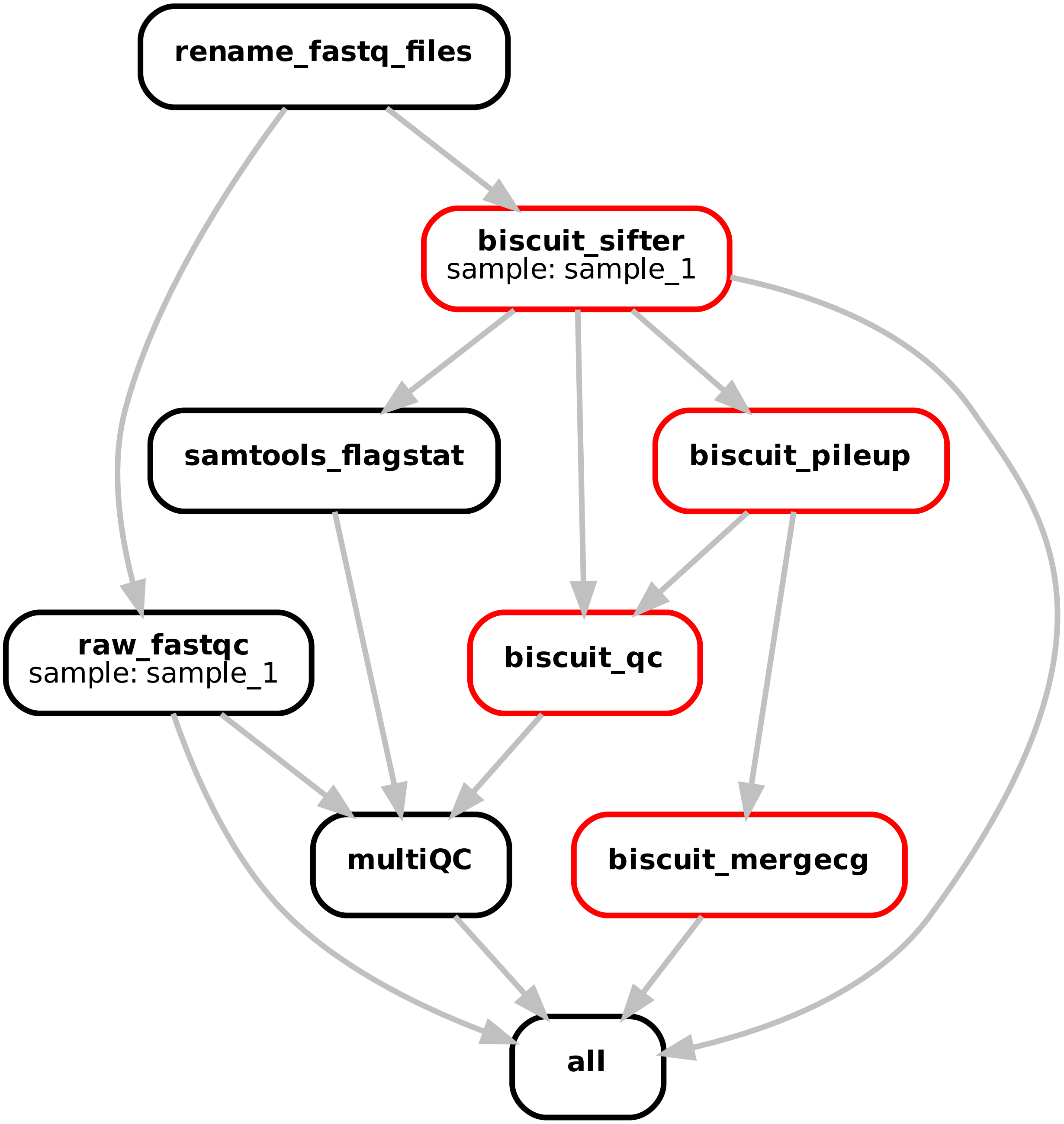

Supplemental Figure S5: **The BISCUIT Snakemake pipeline enables scaling of WGBS processing from a single sample to cohort level analyses.** Directed acyclic graph (DAG) of the default BISCUIT Snakemake workflow. Snakemake is a scalable workflow management system based on named rules represented as nodes. Each rule has a required set of inputs. Most of these inputs are generated by other rules, creating the rule dependencies depicted as directed edges. Steps using BISCUIT are shown in red, while steps using third party tools are shown in black.

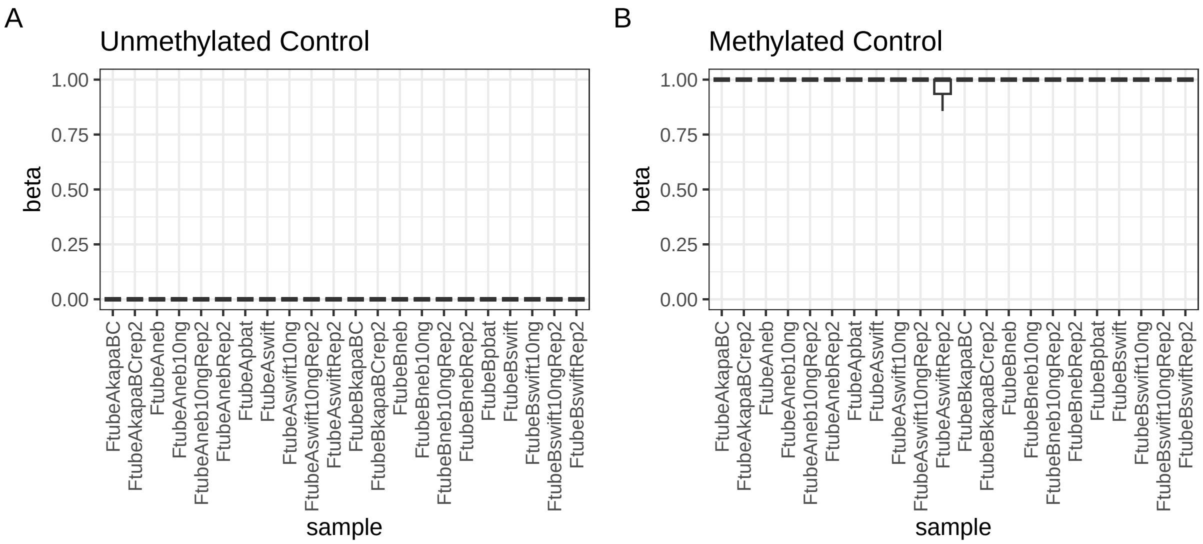

Supplemental Figure S6: **Per-sample methylation control vector plots can be natively produced by the BISCUIT Snakemake pipeline.** Synthetic spike-in controls can provide an orthogonal measure of per-sample conversion rates in WGBS. (A) Unmethylated control (lambda phage) and (B) methylated control (pUC19 vector) beta values (i.e., methylation level) plots reflect the expected methylation states across assayed CpGs. Median methylation for the fully unmethylated spike-in and fully methylated control vector are 0 and 1, respectively. The minimum read depth required is at least 1 read covering a CpG (default in BISCUIT).

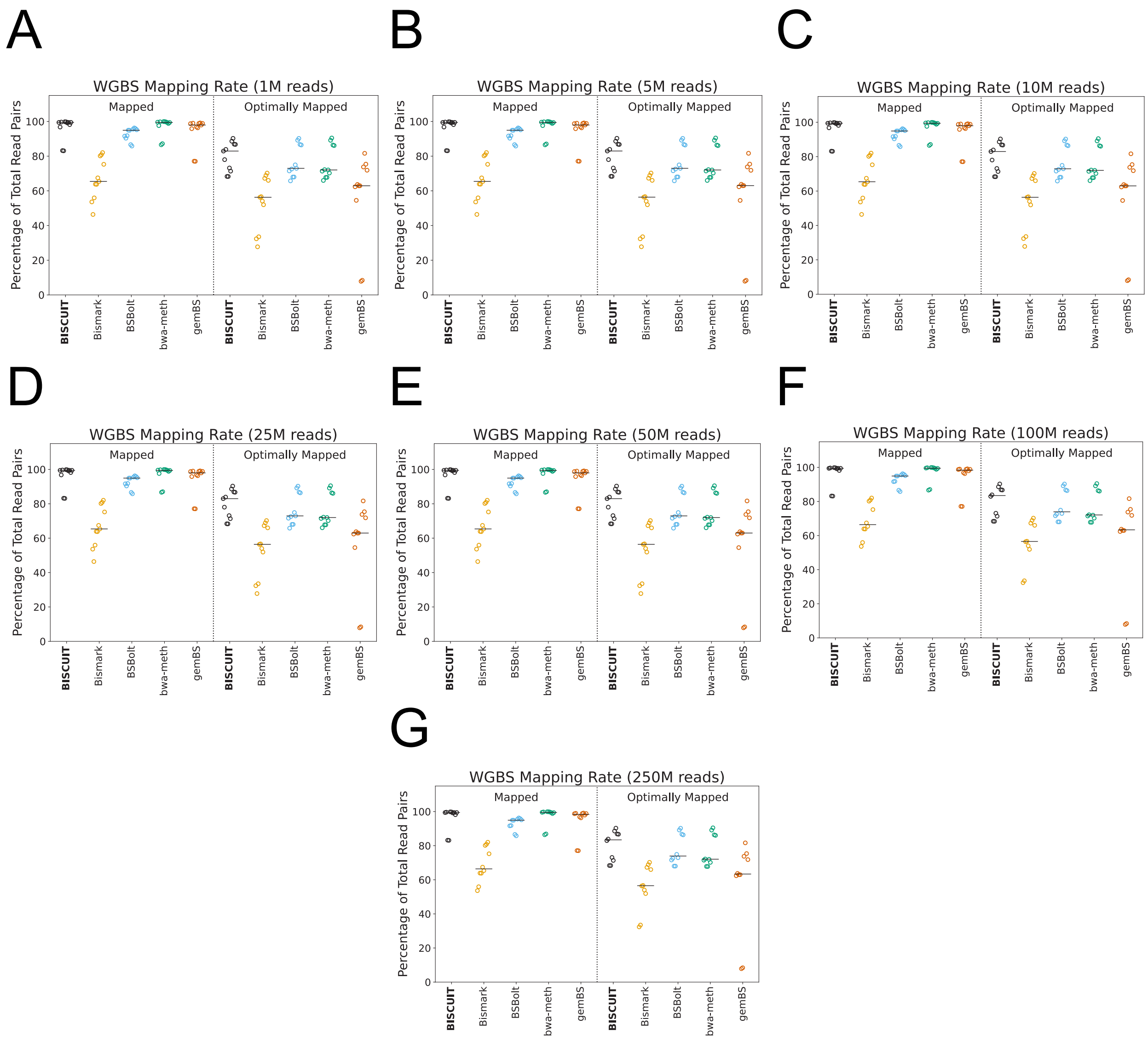

Supplemental Figure S7: **Percentage of mapped and optimally mapped reads across varying read counts for BISCUIT, Bismark, BSBolt, bwa-meth, and gemBS.** (A)–(G) The percentage of reads that were mapped and optimally mapped in Figure 3E. "Mapped" reads includes all primary alignments, whereas "optimally mapped" reads are only primary alignments with a mapping quality (MAPQ) score greater than or equal to 40. Note, Bismark uses a different method for calculating the MAPQ score than the other aligners. Therefore, there is no clear way to define a consistent optimally mapped read across all three aligners.

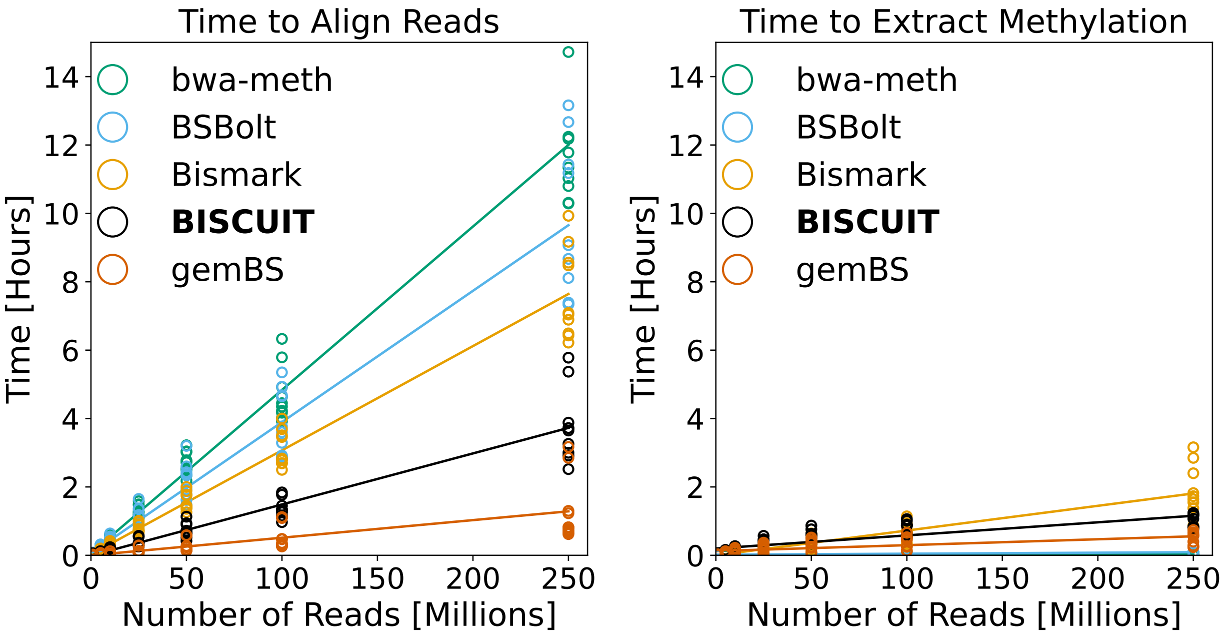

(A) (B)

Supplemental Figure S8: **Comparison of BISCUIT and other cytosine-conversion-aware aligners for aligning reads and extracting methylation.** (A) Alignment + duplicate marking + sorting and indexing (if necessary) time and (B) methylation extraction time across subsampled read depths.

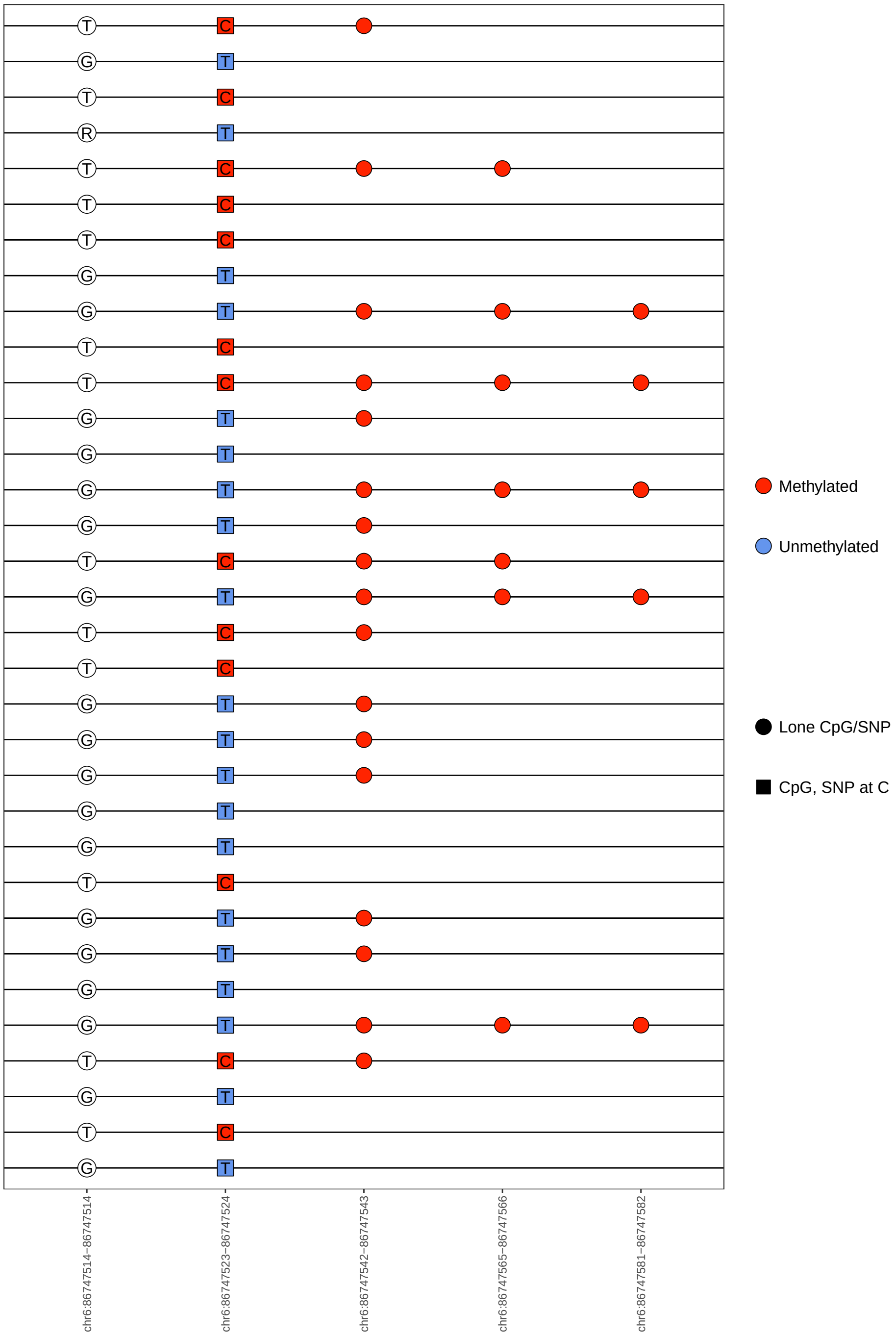

Supplemental Figure S9: **BISCUIT resolves SNP and methylation states in a CpG context.** Here, a SNP and CpG show a statistically significant (Fisher's exact test p-value: 1.2×10^{-9}) match between the SNP base and the CpG methylation state. However, the surrounding region does not show a similar separation in methylation states. Further, a C to T SNP in the cytosine of the CpG dinucleoutide confounds the methylation state, which adds additional complexity to making an allele-specific methylation call at this site.

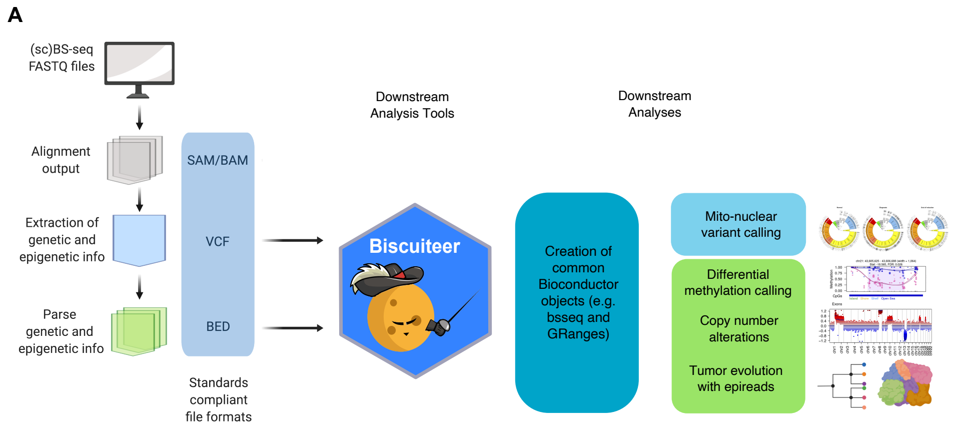

Supplemental Figure S10: **Biscuiteer wrangles BISCUIT output to readily integrate with existing tooling within the Bioconductor analysis ecosystem.** Here, we show a high-level overview of how biscuiteer captures and creates common Bioconductor objects to work with existing downstream tooling for various bisulfite- and non-bisulfite-related sequencing analyses.

References

[1] The ENCODE Project Consortium. “An integrated encyclopedia of DNA elements in the human genome”. en. In: *Nature* 489.7414 (Sept. 2012). Number: 7414 Publisher: Nature Publishing Group, pp. 57–74. ISSN: 1476-4687. DOI: 10.1038/nature11247. URL: https://www.nature.com/articles/nature11247 (visited on 01/23/2023).

[2] Jonathan Foox et al. “The SEQC2 epigenomics quality control (EpiQC) study”. In: *Genome Biology* 22.1 (Dec. 2021), p. 332. ISSN: 1474-760X. DOI: 10.1186/s13059-021-02529-2. URL: https://doi.org/10.1186/s13059-021-02529-2 (visited on 01/23/2023).

[3] Nastaran Heidari et al. “Genome-wide map of regulatory interactions in the human genome”. en. In: *Genome Res.* 24.12 (Dec. 2014). Company: Cold Spring Harbor Laboratory Press Distributor: Cold Spring Harbor Laboratory Press Institution: Cold Spring Harbor Laboratory Press Label: Cold Spring Harbor Laboratory Press Publisher: Cold Spring Harbor Lab, pp. 1905–1917. ISSN: 1088-9051, 1549-5469. DOI: 10.1101/gr.176586.114. URL: https://genome.cshlp.org/content/24/12/1905 (visited on 01/23/2023).

[4] Chongyuan Luo et al. “Robust single-cell DNA methylome profiling with snmC-seq2”. en. In: *Nat Commun* 9.1 (Sept. 2018). Number: 1 Publisher: Nature Publishing Group, p. 3824. ISSN: 2041-1723. DOI: 10.1038/s41467-018-06355-2. URL: https://www.nature.com/articles/s41467-018-06355-2 (visited on 01/23/2023).

[5] Jacob Morrison et al. “Evaluation of whole-genome DNA methylation sequencing library preparation protocols”. In: *Epigenetics & Chromatin* 14.1 (June 2021), p. 28. ISSN: 1756-8935. DOI: 10.1186/s13072-021-00401-y. URL: https://doi.org/10.1186/s13072-021-00401-y (visited on 01/23/2023).

[6] Samuel E. Ross, Daniel Hesselson, and Ozren Bogdanovic. “Developmental Accumulation of Gene Body and Transposon Non-CpG Methylation in the Zebrafish Brain”. In: *Frontiers in Cell and Developmental Biology* 9 (2021). ISSN: 2296-634X. URL: https://www.frontiersin.org/articles/10.3389/fcell.2021.643603 (visited on 01/23/2023).

[7] Samuel E Ross et al. “Developmental remodelling of non-CG methylation at satellite DNA repeats”. In: *Nucleic Acids Research* 48.22 (Dec. 2020), pp. 12675–12688. ISSN: 0305-1048. DOI: 10.1093/nar/gkaa1135. URL: https://doi.org/10.1093/nar/gkaa1135 (visited on 01/23/2023).

[8] Sébastien A. Smallwood et al. “Single-cell genome-wide bisulfite sequencing for assessing epigenetic heterogeneity”. en. In: *Nat Methods* 11.8 (Aug. 2014). Number: 8 Publisher: Nature Publishing Group, pp. 817–820. ISSN: 1548-7105. DOI: 10.1038/nmeth.3035. URL: https://www.nature.com/articles/nmeth.3035 (visited on 01/23/2023).

[9] D. H. Spencer et al. “Epigenomic analysis of the HOX gene loci reveals mechanisms that may control canonical expression patterns in AML and normal hematopoietic cells”. en. In: *Leukemia* 29.6 (June 2015). Number: 6 Publisher: Nature Publishing Group, pp. 1279–1289. ISSN: 1476-5551. DOI: 10.1038/leu.2015.6. URL: https://www.nature.com/articles/leu20156 (visited on 01/23/2023).

[10] Romualdas Vaisvila et al. "Enzymatic methyl sequencing detects DNA methylation at single-base resolution from picograms of DNA". en. In: *Genome Res.* 31.7 (July 2021). Company: Cold Spring Harbor Laboratory Press Distributor: Cold Spring Harbor Laboratory Press Institution: Cold Spring Harbor Laboratory Press Label: Cold Spring Harbor Laboratory Press Publisher: Cold Spring Harbor Lab, pp. 1280–1289. ISSN: 1088-9051, 1549-5469. DOI: 10.1101/gr.266551.120. URL: https://genome.cshlp.org/content/31/7/1280 (visited on 01/23/2023).

[11] Zhen Xu et al. "Zfp57 Exerts Maternal and Sexually Dimorphic Effects on Genomic Imprinting". In: *Frontiers in Cell and Developmental Biology* 10 (2022). ISSN: 2296-634X. URL: https://www.frontiersin.org/articles/10.3389/fcell.2022.784128 (visited on 01/23/2023).

[12] Wanding Zhou et al. "DNA methylation loss in late-replicating domains is linked to mitotic cell division". en. In: *Nat Genet* 50.4 (Apr. 2018). Number: 4 Publisher: Nature Publishing Group, pp. 591–602. ISSN: 1546-1718. DOI: 10.1038/s41588-018-0073-4. URL: https://www.nature.com/articles/s41588-018-0073-4 (visited on 01/23/2023).
